# The effect of LRRK2 loss-of-function variants in humans

**DOI:** 10.1038/s41591-020-0893-5

**Published:** 2020-05-27

**Authors:** Nicola Whiffin, Irina M. Armean, Aaron Kleinman, Jamie L. Marshall, Eric V. Minikel, Julia K. Goodrich, Nicholas M. Quaife, Joanne B. Cole, Qingbo Wang, Konrad J. Karczewski, Beryl B. Cummings, Laurent Francioli, Kristen Laricchia, Anna Guan, Babak Alipanahi, Peter Morrison, Marco A. S. Baptista, Kalpana M. Merchant, Genome Aggregation Database Production Team, Irina M. Armean, Eric Banks, Louis Bergelson, Kristian Cibulskis, Ryan L. Collins, Kristen M. Connolly, Miguel Covarrubias, Beryl Cummings, Mark J. Daly, Stacey Donnelly, Yossi Farjoun, Steven Ferriera, Stacey Gabriel, Laura D. Gauthier, Jeff Gentry, Namrata Gupta, Thibault Jeandet, Diane Kaplan, Kristen M. Laricchia, Christopher Llanwarne, Ruchi Munshi, Benjamin M. Neale, Sam Novod, Anne H. O’Donnell-Luria, Nikelle Petrillo, Timothy Poterba, David Roazen, Valentin Ruano-Rubio, Andrea Saltzman, Kaitlin E. Samocha, Molly Schleicher, Cotton Seed, Matthew Solomonson, Jose Soto, Grace Tiao, Kathleen Tibbetts, Charlotte Tolonen, Christopher Vittal, Gordon Wade, Arcturus Wang, Nicholas A. Watts, Ben Weisburd, Genome Aggregation Database Consortium, Carlos A. Aguilar-Salinas, Tariq Ahmad, Christine M. Albert, Diego Ardissino, Gil Atzmon, John Barnard, Laurent Beaugerie, Emelia J. Benjamin, Michael Boehnke, Lori L. Bonnycastle, Erwin P. Bottinger, Donald W. Bowden, Matthew J. Bown, John C. Chambers, Juliana C. Chan, Daniel Chasman, Judy Cho, Mina K. Chung, Bruce Cohen, Adolfo Correa, Dana Dabelea, Dawood Darbar, Ravindranath Duggirala, Josée Dupuis, Patrick T. Ellinor, Roberto Elosua, Jeanette Erdmann, Martti Färkkilä, Jose Florez, Andre Franke, Gad Getz, Benjamin Glaser, Stephen J. Glatt, David Goldstein, Clicerio Gonzalez, Leif Groop, Christopher Haiman, Craig Hanis, Matthew Harms, Mikko Hiltunen, Matti M. Holi, Christina M. Hultman, Mikko Kallela, Jaakko Kaprio, Sekar Kathiresan, Bong-Jo Kim, Young Jin Kim, George Kirov, Jaspal Kooner, Seppo Koskinen, Harlan M. Krumholz, Subra Kugathasan, Soo Heon Kwak, Markku Laakso, Terho Lehtimäki, Ruth J. F. Loos, Steven A. Lubitz, Ronald C. W. Ma, Daniel G. MacArthur, Jaume Marrugat, Kari M. Mattila, Steven McCarroll, Mark I. McCarthy, Dermot McGovern, Ruth McPherson, James B. Meigs, Olle Melander, Andres Metspalu, Peter M. Nilsson, Michael C. O’Donovan, Dost Ongur, Lorena Orozco, Michael J. Owen, Colin N. A. Palmer, Aarno Palotie, Kyong Soo Park, Carlos Pato, Ann E. Pulver, Nazneen Rahman, Anne M. Remes, John D. Riou, Samuli Ripatti, Dan M. Roden, Danish Saleheen, Veikko Salomaa, Nilesh J. Samani, Jeremiah Scharf, Heribert Schunkert, Moore B. Shoemaker, Pamela Sklar, Hilkka Soininen, Harry Sokol, Tim Spector, Patrick F. Sullivan, Jaana Suvisaari, E. Shyong Tai, Yik Ying Teo, Tuomi Tiinamaija, Ming Tsuang, Dan Turner, Teresa Tusie-Luna, Erkki Vartiainen, Marquis P. Vawter, James S. Ware, Hugh Watkins, Rinse K. Weersma, Maija Wessman, James G. Wilson, Ramnik J. Xavier, James S. Ware, Aki S. Havulinna, Bozenna Iliadou, Jung-Jin Lee, Girish N. Nadkarni, Cole Whiteman, 23andMe Research Team, Michelle Agee, Adam Auton, Robert K. Bell, Katarzyna Bryc, Sarah L. Elson, Pierre Fontanillas, Nicholas A. Furlotte, Barry Hicks, David A. Hinds, Karen E. Huber, Ethan M. Jewett, Yunxuan Jiang, Keng-Han Lin, Nadia K. Litterman, Matthew H. McIntyre, Kimberly F. McManus, Joanna L. Mountain, Elizabeth S. Noblin, Carrie A. M. Northover, Steven J. Pitts, G. David Poznik, J. Fah Sathirapongsasuti, Janie F. Shelton, Suyash Shringarpure, Chao Tian, Joyce Y. Tung, Vladimir Vacic, Xin Wang, Catherine H. Wilson, Mark Daly, Tõnu Esko, Christina Hultman, Ruth J. F. Loos, Lili Milani, Aarno Palotie, Carlos Pato, Michele Pato, Danish Saleheen, Patrick F. Sullivan, Jessica Alföldi, Paul Cannon, Daniel G. MacArthur

**Affiliations:** 1 National Heart & Lung Institute and MRC London Institute of Medical Sciences, Imperial College London, London, UK; 2 Cardiovascular Research Centre, Royal Brompton & Harefield Hospitals NHS Trust, London, UK; 3 Program in Medical and Population Genetics, Broad Institute of MIT and Harvard, Cambridge, MA USA; 4 Analytic and Translational Genetics Unit, Massachusetts General Hospital, Boston, MA USA; 5 23andMe, Inc., Sunnyvale, CA USA; 6 Program in Metabolism, Broad Institute of MIT and Harvard, Cambridge, MA USA; 7 Center for Genomic Medicine, Massachusetts General Hospital, Boston, MA USA; 8 Division of Endocrinology and Center for Basic and Translational Obesity Research, Boston Children’s Hospital, Boston, MA USA; 9 Program in Bioinformatics and Integrative Genomics, Harvard Medical School, Boston, MA USA; 10 Program in Biological and Biomedical Sciences, Harvard Medical School, Boston, MA USA; 11 Michael J. Fox Foundation, New York, NY USA; 12 National Institute for Health and Welfare, Helsinki, Finland; 13 Institute for Molecular Medicine Finland (FIMM), HiLIFE, University of Helsinki, Helsinki, Finland; 14 Department of Medical Epidemiology and Biostatistics, Karolinska Institutet, Stockholm, Sweden; 15 Department of Biostatistics and Epidemiology, Perelman School of Medicine at the University of Pennsylvania, Philadelphia, PA USA; 16 The Charles Bronfman Institute for Personalized Medicine, Icahn School of Medicine at Mount Sinai, New York, NY USA; 17 Department of Medicine, Icahn School of Medicine at Mount Sinai, New York, NY USA; 18 Department of Psychiatry and the Behavioral Sciences, State University of New York, Downstate Medical Center, New York, NY USA; 19 Stanley Center for Psychiatric Research, Broad Institute of MIT and Harvard, Cambridge, MA USA; 20 Estonian Genome Center, Institute of Genomics, University of Tartu, Tartu, Estonia; 21 The Mindich Child Health and Development Institute, Icahn School of Medicine at Mount Sinai, New York, NY USA; 22 Department of Medicine, Perelman School of Medicine at the University of Pennsylvania, Philadelphia, PA USA; 23 Center for Non-Communicable Diseases, Karachi, Pakistan; 24 Departments of Genetics and Psychiatry, University of North Carolina, Chapel Hill, NC, USA; 25 Present Address: Google, Inc., Mountain View, CA USA; 26 Present Address: Centre for Population Genomics, Garvan Institute of Medical Research, and UNSW Sydney, Sydney, New South Wales Australia; 27 Present Address: Centre for Population Genomics, Murdoch Children’s Research Institute, Melbourne, Victoria, Australia; 28 European Molecular Biology Laboratory, European Bioinformatics Institute, Wellcome Genome Campus, Hinxton, UK; 29 Data Sciences Platform, Broad Institute of MIT and Harvard, Cambridge, MA USA; 30 Genomics Platform, Broad Institute of MIT and Harvard, Cambridge, MA USA; 31 Broad Genomics, Broad Institute of MIT and Harvard, Cambridge, MA USA; 32 Division of Genetics and Genomics, Boston Children’s Hospital, Boston, MA USA; 33 Department of Pediatrics, Harvard Medical School, Boston, MA USA; 34 Wellcome Sanger Institute, Wellcome Genome Campus, Hinxton, UK; 35 Unidad de Investigacion de Enfermedades Metabolicas, Instituto Nacional de Ciencias Medicas y Nutricion, Mexico City, Mexico; 36 Peninsula College of Medicine and Dentistry, Exeter, UK; 37 Division of Preventive Medicine, Brigham and Women’s Hospital, Boston, MA USA; 38 Division of Cardiovascular Medicine, Brigham and Women’s Hospital and Harvard Medical School, Boston, MA USA; 39 Department of Cardiology, University Hospital, Parma, Italy; 40 Department of Biology, Faculty of Natural Sciences, University of Haifa, Haifa, Israel; 41 Departments of Medicine and Genetics, Albert Einstein College of Medicine, Bronx, NY USA; 42 Department of Quantitative Health Sciences, Lerner Research Institute, Cleveland Clinic, Cleveland, OH USA; 43 Gastroenterology Department, Sorbonne Université, APHP, Saint Antoine Hospital, Paris, France; 44 NHLBI and Boston University’s Framingham Heart Study, Framingham, MA USA; 45 Department of Medicine, Boston University School of Medicine, Boston, MA USA; 46 Department of Epidemiology, Boston University School of Public Health, Boston, MA USA; 47 Department of Biostatistics and Center for Statistical Genetics, University of Michigan, Ann Arbor, MI USA; 48 National Human Genome Research Institute, National Institutes of Health, Bethesda, MD USA; 49 Department of Biochemistry, Wake Forest School of Medicine, Winston-Salem, NC USA; 50 Center for Genomics and Personalized Medicine Research, Wake Forest School of Medicine, Winston-Salem, NC USA; 51 Center for Diabetes Research, Wake Forest School of Medicine, Winston-Salem, NC USA; 52 Department of Cardiovascular Sciences, University of Leicester, Leicester, UK; 53 NIHR Leicester Biomedical Research Centre, Glenfield Hospital, Leicester, UK; 54 Department of Epidemiology and Biostatistics, Imperial College London, London, UK; 55 Department of Cardiology, Ealing Hospital NHS Trust, Southall, UK; 56 Imperial College Healthcare NHS Trust, Imperial College London, London, UK; 57 Department of Medicine and Therapeutics, The Chinese University of Hong Kong, Hong Kong, China; 58 Department of Medicine, Harvard Medical School, Boston, MA USA; 59 Departments of Cardiovascular Medicine Cellular and Molecular Medicine, Molecular Cardiology, and Quantitative Health Sciences, Cleveland Clinic, Cleveland, OH USA; 60 McLean Hospital, Belmont, MA USA; 61 Department of Medicine, University of Mississippi Medical Center, Jackson, MS USA; 62 Department of Epidemiology, Colorado School of Public Health, Aurora, CO USA; 63 Department of Medicine and Pharmacology, University of Illinois at Chicago, Champaign, IL USA; 64 Department of Genetics, Texas Biomedical Research Institute, San Antonio, TX USA; 65 Department of Biostatistics, Boston University School of Public Health, Boston, MA USA; 66 Framingham Heart Study, National Heart, Lung and Blood Institute, Framingham, MA USA; 67 Cardiac Arrhythmia Service and Cardiovascular Research Center, Massachusetts General Hospital, Boston, MA USA; 68 Cardiovascular Epidemiology and Genetics, Hospital del Mar Medical Research Institute (IMIM), Barcelona, Spain; 69 CIBER CV, Barcelona, Spain; 70 Department of Medicine, Medical School, University of Vic-Central University of Catalonia, Vic, Spain; 71 Institute for Cardiogenetics, University of Lübeck, Lübeck, Germany; 72 DZHK (German Research Centre for Cardiovascular Research), Partner Site Hamburg/Lübeck/Kiel, Lübeck, Germany; 73 University Heart Center Lübeck, Lübeck, Germany; 74 Clinic of Gastroenterology, Helsinki University and Helsinki University Hospital, Helsinki, Finland; 75 Diabetes Unit and Center for Genomic Medicine, Massachusetts General Hospital; Programs in Metabolism and Medical & Population Genetics, Broad Institute; Department of Medicine, Harvard Medical School, Boston, MA USA; 76 Institute of Clinical Molecular Biology (IKMB), Christian-Albrechts University of Kiel, Kiel, Germany; 77 Bioinformatics Program, MGH Cancer Center and Department of Pathology, Boston, MA USA; 78 Cancer Genome Computational Analysis, Broad Institute, Cambridge, MA USA; 79 Endocrinology and Metabolism Department, Hadassah-Hebrew University Medical Center, Jerusalem, Israel; 80 Department of Psychiatry and Behavioral Sciences, SUNY Upstate Medical University, Syracuse, NY USA; 81 Institute for Genomic Medicine, Columbia University Medical Center, Hammer Health Sciences, New York, NY USA; 82 Department of Genetics & Development, Columbia University Medical Center, Hammer Health Sciences, New York, NY USA; 83 Centro de Investigacion en Salud Poblacional, Instituto Nacional de Salud Publica Mexico, Cuernavaca, Mexico; 84 Lund University, Lund, Sweden; 85 Lund University Diabetes Centre, Lund, Sweden; 86 Human Genetics Center, University of Texas Health Science Center at Houston, Houston, TX USA; 87 Department of Neurology, Columbia University, New York, NY USA; 88 Institute of Biomedicine, University of Eastern Finland, Kuopio, Finland; 89 Department of Psychiatry, PL 320, Helsinki University Central Hospital, Lapinlahdentie, Helsinki, Finland; 90 Icahn School of Medicine at Mount Sinai, New York, NY USA; 91 Department of Neurology, Helsinki University Central Hospital, Helsinki, Finland; 92 Department of Public Health, Faculty of Medicine, University of Helsinki, Helsinki, Finland; 93 Cardiovascular Disease Initiative and Program in Medical and Population Genetics, Broad Institute of MIT and Harvard, Cambridge, MA USA; 94 Center for Genome Science, Korea National Institute of Health, Chungcheongbuk-do, Republic of Korea; 95 MRC Centre for Neuropsychiatric Genetics & Genomics, Cardiff University School of Medicine, Cardiff, UK; 96 National Heart and Lung Institute, Cardiovascular Sciences, Hammersmith Campus, Imperial College London, London, UK; 97 Department of Health, THL-National Institute for Health and Welfare, Helsinki, Finland; 98 Section of Cardiovascular Medicine, Department of Internal Medicine, Yale School of Medicine, New Haven, CT USA; 99 Center for Outcomes Research and Evaluation, Yale-New Haven Hospital, New Haven, CT USA; 100 Division of Pediatric Gastroenterology, Emory University School of Medicine, Atlanta, GA USA; 101 Department of Internal Medicine, Seoul National University Hospital, Seoul, Republic of Korea; 102 Institute of Clinical Medicine, The University of Eastern Finland, Kuopio, Finland; 103 Kuopio University Hospital, Kuopio, Finland; 104 Department of Clinical Chemistry, Fimlab Laboratories and Finnish Cardiovascular Research Center-Tampere, Faculty of Medicine and Health Technology, Tampere University, Tampere, Finland; 105 Li Ka Shing Institute of Health Sciences, The Chinese University of Hong Kong, Hong Kong, China; 106 Hong Kong Institute of Diabetes and Obesity, The Chinese University of Hong Kong, Hong Kong, China; 107 Cardiovascular Research REGICOR Group, Hospital del Mar Medical Research Institute (IMIM), Barcelona, Spain; 108 Department of Genetics, Harvard Medical School, Boston, MA USA; 109 Oxford Centre for Diabetes, Endocrinology and Metabolism, University of Oxford, Churchill Hospital, Oxford, UK; 110 Wellcome Centre for Human Genetics, University of Oxford, Oxford, UK; 111 Oxford NIHR Biomedical Research Centre, Oxford University Hospitals NHS Foundation Trust, John Radcliffe Hospital, Oxford, UK; 112 F Widjaja Foundation Inflammatory Bowel and Immunobiology Research Institute, Cedars-Sinai Medical Center, Los Angeles, CA USA; 113 Atherogenomics Laboratory, University of Ottawa Heart Institute, Ottawa, Ontario Canada; 114 Division of General Internal Medicine, Massachusetts General Hospital, Boston, MA USA; 115 Department of Clinical Sciences, University Hospital Malmo Clinical Research Center, Lund University, Malmo, Sweden; 116 Department of Clinical Sciences, Lund University, Skane University Hospital, Malmo, Sweden; 117 Instituto Nacional de Medicina Genómica (INMEGEN), Mexico City, Mexico; 118 Medical Research Institute, Ninewells Hospital and Medical School, University of Dundee, Dundee, UK; 119 Department of Molecular Medicine and Biopharmaceutical Sciences, Graduate School of Convergence Science and Technology, Seoul National University, Seoul, Republic of Korea; 120 Department of Psychiatry, Keck School of Medicine at the University of Southern California, Los Angeles, CA USA; 121 Department of Psychiatry and Behavioral Sciences, Johns Hopkins University School of Medicine, Baltimore, MD USA; 122 Division of Genetics and Epidemiology, Institute of Cancer Research, London, UK; 123 Medical Research Center, Oulu University Hospital, Oulu, Finland and Research Unit of Clinical Neuroscience, Neurology, University of Oulu, Oulu, Finland; 124 Research Center, Montreal Heart Institute, Montreal, Québec Canada; 125 Department of Medicine, Faculty of Medicine, Université de Montréal, Montreal, Québec Canada; 126 Broad Institute of MIT and Harvard, Cambridge, MA USA; 127 Department of Biomedical Informatics, Vanderbilt University Medical Center, Nashville, TN USA; 128 Department of Medicine, Vanderbilt University Medical Center, Nashville, TN USA; 129 Deutsches Herzzentrum München, München, Germany; 130 Technische Universität München, München, Germany; 131 Division of Cardiovascular Medicine, School of Medicine, Nashville VA Medical Center and Vanderbilt University, Nashville, TN USA; 132 Department of Psychiatry, Icahn School of Medicine at Mount Sinai, New York, NY USA; 133 Department of Genetics and Genomic Sciences, Icahn School of Medicine at Mount Sinai, New York, NY USA; 134 Institute for Genomics and Multiscale Biology, Icahn School of Medicine at Mount Sinai, New York, NY USA; 135 Department of Twin Research and Genetic Epidemiology, King’s College London, London, UK; 136 Departments of Genetics and Psychiatry, University of North Carolina, Chapel Hill, NC USA; 137 Saw Swee Hock School of Public Health, National University of Singapore, National University Health System, Singapore, Singapore; 138 Department of Medicine, Yong Loo Lin School of Medicine, National University of Singapore, Singapore, Singapore; 139 Duke-NUS Graduate Medical School, Singapore, Singapore; 140 Life Sciences Institute, National University of Singapore, Singapore, Singapore; 141 Department of Statistics and Applied Probability, National University of Singapore, Singapore, Singapore; 142 Folkhälsan Institute of Genetics, Folkhälsan Research Center, Helsinki, Finland; 143 HUCH Abdominal Center, Helsinki University Hospital, Helsinki, Finland; 144 Center for Behavioral Genomics, Department of Psychiatry, University of California, San Diego, CA USA; 145 Institute of Genomic Medicine, University of California, San Diego, CA USA; 146 Juliet Keidan Institute of Pediatric Gastroenterology, Shaare Zedek Medical Center, The Hebrew University of Jerusalem, Jerusalem, Israel; 147 Instituto de Investigaciones Biomédicas, UNAM Mexico City, Mexico City, Mexico; 148 Instituto Nacional de Ciencias Médicas y Nutrición, Salvador Zubirán Mexico City, Mexico City, Mexico; 149 Department of Psychiatry & Human Behavior, University of California Irvine, Irvine, CA USA; 150 Radcliffe Department of Medicine, University of Oxford, Oxford, UK; 151 Department of Gastroenterology and Hepatology, University of Groningen and University Medical Center Groningen, Groningen, the Netherlands; 152 Department of Physiology and Biophysics, University of Mississippi Medical Center, Jackson, MS USA; 153 Program in Infectious Disease and Microbiome, Broad Institute of MIT and Harvard, Cambridge, MA USA; 154 Center for Computational and Integrative Biology, Massachusetts General Hospital, Boston, MA USA

**Keywords:** Genomics, Neurological disorders

## Abstract

Human genetic variants predicted to cause loss-of-function of protein-coding genes (pLoF variants) provide natural in vivo models of human gene inactivation and can be valuable indicators of gene function and the potential toxicity of therapeutic inhibitors targeting these genes^1,2^. Gain-of-kinase-function variants in *LRRK2* are known to significantly increase the risk of Parkinson’s disease^3,4^, suggesting that inhibition of LRRK2 kinase activity is a promising therapeutic strategy. While preclinical studies in model organisms have raised some on-target toxicity concerns^5–8^, the biological consequences of LRRK2 inhibition have not been well characterized in humans. Here, we systematically analyze pLoF variants in *LRRK2* observed across 141,456 individuals sequenced in the Genome Aggregation Database (gnomAD)^9^, 49,960 exome-sequenced individuals from the UK Biobank and over 4 million participants in the 23andMe genotyped dataset. After stringent variant curation, we identify 1,455 individuals with high-confidence pLoF variants in *LRRK2*. Experimental validation of three variants, combined with previous work^10^, confirmed reduced protein levels in 82.5% of our cohort. We show that heterozygous pLoF variants in *LRRK2* reduce LRRK2 protein levels but that these are not strongly associated with any specific phenotype or disease state. Our results demonstrate the value of large-scale genomic databases and phenotyping of human loss-of-function carriers for target validation in drug discovery.

## Abstract (web summary)

Analysis of large genomic datasets, including gnomAD, reveals that partial *LRRK2* loss of function is not strongly associated with diseases, serving as an example of how human genetics can be leveraged for target validation in drug discovery.

## Main

New therapeutic strategies are desperately needed in Parkinson’s disease (PD), one of the most common age-related neurological diseases, which affects about 1% of people over the age of 60 years^11,12^. Around 30% of familial and 3–5% of sporadic PD cases have been linked to a genetic cause^13^. *LRRK2* missense variants account for a large fraction of cases, including high-penetrance variants^14^, moderately penetrant variants such as G2019S^15^ and risk factors identified in genome-wide association studies^16^. Although the precise mechanism by which *LRRK2* variants mediate their pathogenicity remains unclear, a common feature is augmentation of kinase activity associated with disease-relevant alterations in cell models^3,17,18^. Discovery of Rab GTPases as LRRK2 (ref. ^19^) substrates highlighted the role of LRRK2 in regulation of the endolysosomal and vesicular trafficking pathways implicated in PD^19,20^. LRRK2 kinase activity is also upregulated more generally in patients with PD (with and without LRRK2 variants)^21^. LRRK2 has therefore become a prominent drug target, with multiple LRRK2 kinase inhibitors and suppressors^22^ in development as disease-modifying treatments for PD^21,23,24^. There are three LRRK2 therapeutics currently in early clinical testing from both Denali (small molecules DNL201, ClinicalTrials.gov Identifier: NCT03710707 and DNL151, ClinicalTrials.gov Identifier: NCT04056689) and Biogen (antisense oligonucleotide BIIB094, ClinicalTrials.gov Identifier: NCT03976349).

Despite these promising indications, there are concerns about the potential toxicity of LRRK2 inhibitors. These mainly arise from preclinical studies, where homozygous knockouts of *LRRK2* in mice and high-dose toxicology studies of LRRK2 kinase inhibitors in rats and primates, have shown abnormal phenotypes in the lung, kidney and liver^5–8^. While model organisms are invaluable for understanding the function of LRRK2, they also have important limitations, as exemplified by inconsistencies in phenotypic consequences of reduced LRRK2 activity seen among yeast, fruit flies, worms, mice, rats and nonhuman primates^25^. Complementary data from natural human knockouts are critical for understanding both gene function and the potential consequences of long-term reduction of LRRK2 in humans.

Large-scale human genetics is an increasingly powerful source of data for the discovery and validation of therapeutic targets in humans^1^. pLoF variants, predicted to largely or entirely abolish the function of affected alleles, are a particularly informative class of genetic variation. Such variants are natural models for lifelong organism-wide inhibition of the target gene and can provide information about both the efficacy and safety of a candidate target^2,26–29^. However, pLoF variants are rare in human populations^30^ and are also enriched for both sequencing and annotation artefacts^31^. As such, leveraging pLoF variation in drug target assessment typically requires very large collections of genetically and phenotypically characterized individuals, combined with deep curation of the target gene and candidate variants^32^. Although previous studies of pLoF variants in *LRRK2* have found no association with risk of PD^10^, no study has assessed their broader phenotypic consequences.

We identified *LRRK2* pLoF variants and assessed associated phenotypic changes in three large cohorts of genetically characterized individuals. First, we annotated *LRRK2* pLoF variants in two large sequencing cohorts: the gnomAD v.2.1.1 dataset, which contains 125,748 exomes and 15,708 genomes from unrelated individuals^9^ and 46,062 exome-sequenced unrelated European individuals from the UK Biobank^33^. We identified 633 individuals in gnomAD and 258 individuals in the UK Biobank with 150 unique candidate *LRRK2* loss-of-function (LoF) variants, a combined carrier frequency of 0.48%. All variants were observed only in the heterozygous state. Compared to the spectrum observed across all genes, *LRRK2* is not significantly depleted for pLoF variants in gnomAD (LoF observed/expected upper bound fraction^9^ = 0.64).

**Fig. 1:**
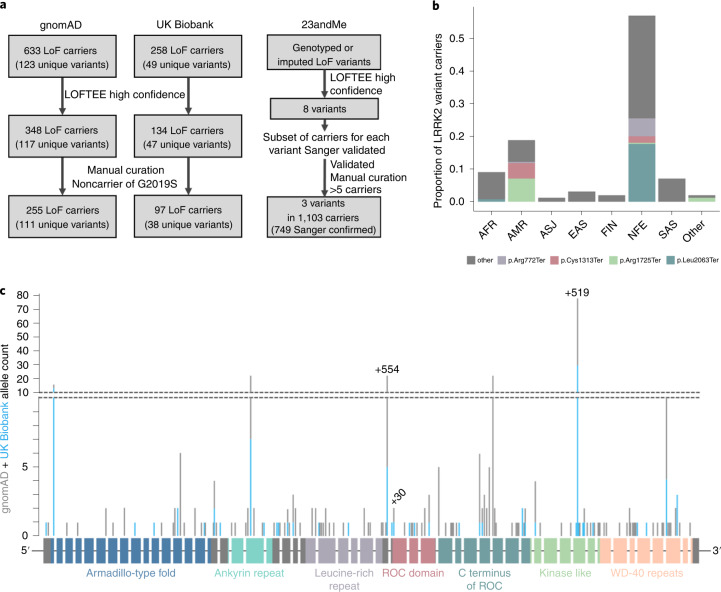
Annotation and curation of candidate *LRRK2* pLoF variants. **a**, Flow chart showing the variant filtering and curation of candidate *LRRK2* LoF variants in the gnomAD, UK Biobank and 23andMe cohorts. Of the 1,103 carriers identified in 23andMe, 749 were confirmed by Sanger sequencing with the remainder untested. **b**, The ancestry distribution of *LRRK2* pLoF variant carriers in gnomAD. AFR, African/African American; AMR, American/Latino; ASJ, Ashkenazi Jewish; EAS, East Asian; FIN, Finnish; NFE, non-Finnish European; SAS, South Asian. The pLoF variants seen more than ten times appear in color with remaining variants in gray. *LRRK2* pLoF variants are mostly individually extremely rare (less than 1 in 10,000 carrier frequency), with the exception of two nonsense variants almost exclusively restricted to the admixed AMR population (Cys1313Ter and Arg1725Ter) and two largely NFE-specific variants (Leu2063Ter and Arg772Ter). All variant protein descriptions are with respect to ENSP00000298910.7. **c**, Schematic of the LRRK2 gene with pLoF variants marked by position, with the height of the marker corresponding to allele count in gnomAD (gray bars) and UK Biobank (blue bars). The 51 exons are shown as rectangles colored by protein domain, with the remaining exons in gray. The three variants genotyped in the 23andMe cohort are annotated with their sample count in black text.

We manually curated the 150 identified variants to remove those of low quality or with annotation errors suggesting that they are unlikely to cause true LoF (Fig. 1a and Supplementary Tables 1 and 2). We removed 16 variants identified as low confidence by the LoF transcript effect estimator ((LOFTEE); 6 variants in 409 individuals)^9^ or manually curated as low quality or unlikely to cause LoF (10 variants in 129 individuals). One additional individual was excluded from the UK Biobank cohort as they carried both a pLoF variant and the G2019S risk allele.

Our final dataset comprised 255 gnomAD individuals and 97 UK Biobank individuals with 134 unique high-confidence pLoF variants (Fig. 1a) and an overall carrier frequency of 0.19%; less than half the frequency estimated from uncurated variants, reaffirming the importance of thorough curation of candidate LoF variants^32^. A subset of 25 gnomAD samples with 19 unique *LRRK2* pLoF variants with DNA available were all successfully validated by Sanger sequencing (Supplementary Table 3).

Second, we examined *LRRK2* pLoF variants in over 4 million consented and array-genotyped research participants from the personal genetics company 23andMe. Eight putative (LOFTEE high confidence) *LRRK2* LoF variants were identified. After manual curation, all putative carriers of each variant were submitted for validation by Sanger sequencing and variants with <5 confirmed carriers were excluded. The resulting cohort comprised 749 individuals, each a Sanger-confirmed carrier for one of three pLoF variants (Fig. 1a and Supplementary Table 4). The high rate of Sanger confirmation for rs183902574 (>98%) allowed confident addition of 354 putative carriers of rs183902574, from expansion of the 23andMe dataset, without Sanger confirmation. Analyses with and without these genotyped-only carriers were not significantly different (Supplementary Table 5). Across the two most frequent pLoF alleles we observed an extremely small number (<5) of sequence-confirmed homozygotes; however, given the very small number of observations, we can make no robust inference, except that homozygous inactivation of LRRK2 seems compatible with life. For the remainder of this manuscript we focus on heterozygous pLoF carriers.

**Extended Data Fig. 1:**
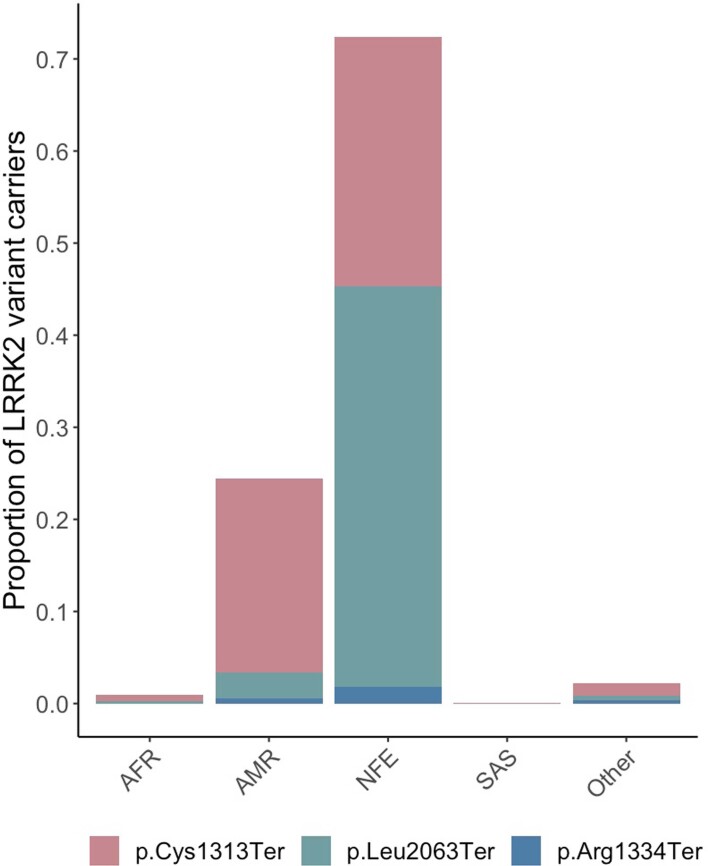
Ethnicity distribution of LRRK2 LoF carriers in the 23andMe cohort. Bars are coloured according to the detected variant. AFR, African/African American; AMR, American/Latino; NFE, non-Finnish European; SAS, South Asian.

The three combined datasets provide a total of 1,455 carrier individuals with 134 unique *LRRK2* pLoF variants. These variants are found across all major continental populations (Fig. 1b and Extended Data Fig. 1) and show neither any obvious clustering along the length of the LRRK2 protein, nor specific enrichment or depletion in any of the known annotated protein domains (chi squared *P* = 0.22; Fig. 1c), consistently with signatures of true LoF^32^.

**Fig. 2:**
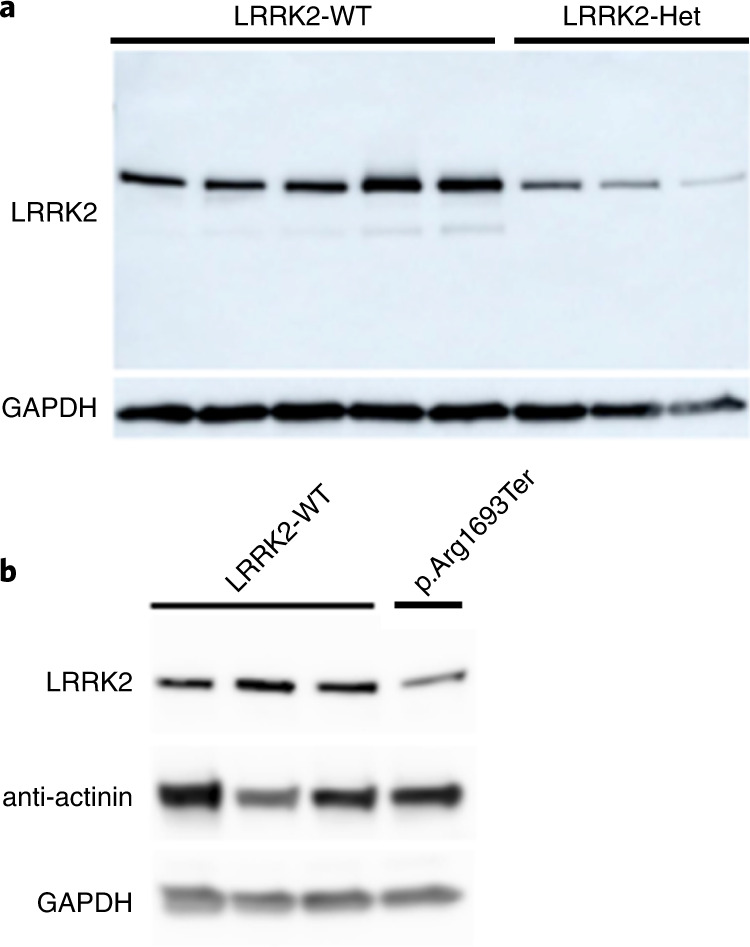
*LRRK2* pLoF heterozygotes have reduced LRRK2 protein compared to cells harboring no LoF variants. **a**, Immunoblot of LRRK2 and loading control GAPDH on LCLs from five individuals harboring no pLoF variants (LRRK2-WT) and three individuals harboring a heterozygous (Het) pLoF variant (Cys1313Ter; 12-40699748-T-A; Arg1483Ter; 12-40704362-C-T). Experiments were repeated ten times with similar results. **b**, Immunoblot of LRRK2, alpha-actinin (specific to muscle) and GAPDH on three control lines and one CRISPR/Cas9-engineered LRRK2 heterozygous line of cardiomyocytes differentiated from embryonic stem cells (ESCs) (Arg1693Ter-12-40714897-C-T). All variant protein descriptions are with respect to ENSP00000298910.7. Experiments were repeated five times with similar results. Source data

**Extended Data Fig. 2:**
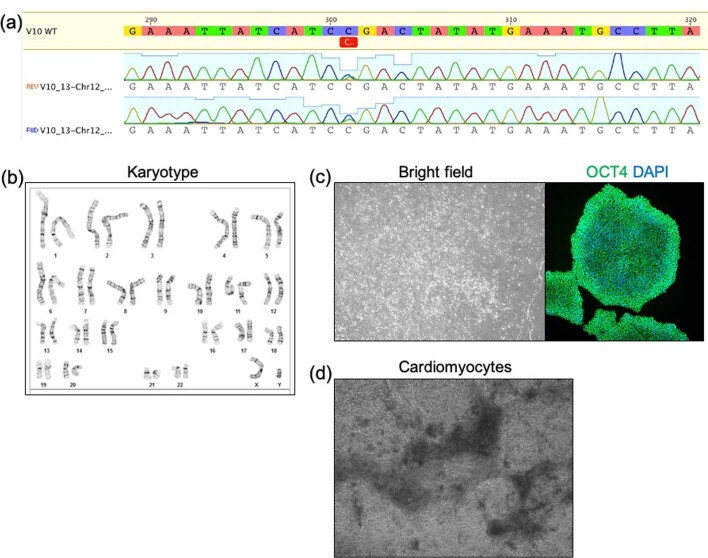
Details of CRISPR/Cas9-engineered embryonic stem cells and cardiomyocyte differentiation. **a**, Sanger sequence of isogenic hESC engineered colony for heterozygous LRRK2 variant 10, clone 13 (GRCh37:12-40714897-C-T). The engineered cell line maintains **b**, a normal karyotype, **c**, normal colony morphology and expression of OCT4, and **d**, differentiates into the cardiomyocyte lineage. The bright field image of cardiomyocytes was captured at day 17 of the differentiation protocol. The cardiomyocyte differentiation was repeated 12 times and the staining for Oct4 was repeated on 30 independent colonies.

To confirm that *LRRK2* pLoF variants result in reduced LRRK2 protein levels, we analyzed total protein lysates from cell lines with three unique pLoF variants. We obtained lymphoblastoid cell lines (LCLs) from two families with naturally occurring heterozygous LoF variants and a third variant was CRISPR/Cas9-engineered into embryonic stem cells (Extended Data Fig. 2), which were differentiated into cardiomyocytes. In all instances, LRRK2 protein levels were visibly reduced compared to noncarrier controls (Fig. 2). These results agree with a previous study which assessed three separate pLoF variants and found significantly reduced LRRK2 protein levels^10^. Together, these six functionally validated variants represent 82.5% of pLoF carriers in this study (1,201 of 1,455). Although heterozygous pLoF carriers have LRRK2 protein remaining, we believe that this state represents a plausible genetic model for therapeutic inhibition of LRRK2, as target engagement by pharmacological inhibitors is unlikely to be complete.

**Fig. 3:**
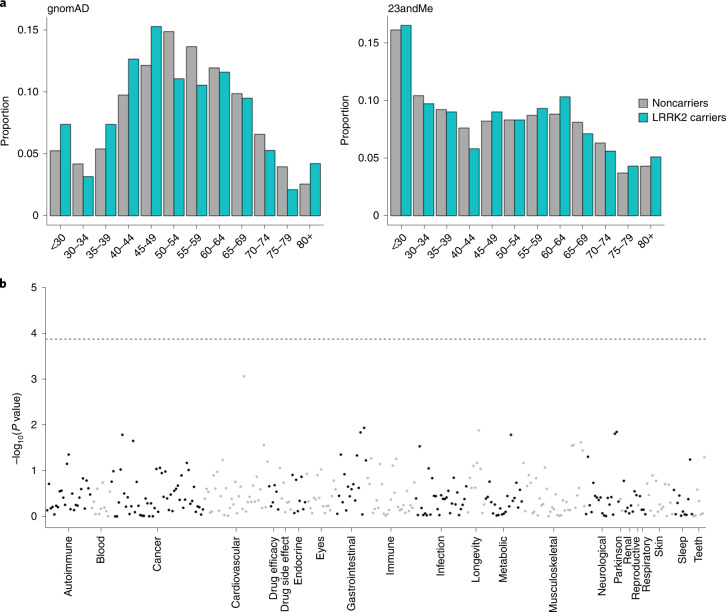
*LRRK2* pLoF variants are not strongly associated with either age distribution or any adverse phenotypes. **a**, The age distributions of *LRRK2* pLoF carriers are not significantly different from those of noncarriers in both gnomAD and 23andMe. Note that this analysis is based on age at sample collection. **b**, Manhattan plot of phenome-wide association study results for carriers of three *LRRK2* pLoF variants against noncarriers in the 23andMe cohort. Each point represents a distinct phenotype, with these grouped into related categories (delineated by alternating black and gray points). The dotted horizontal line represents a Bonferroni-corrected *P* value threshold for 366 tests. Logistic regression was used for binary phenotypes and linear regression for quantitative phenotypes controlling for age, sex, genotyping platform and the first ten genetic principal components. Full association statistics are listed in Supplementary Table 8.

We next sought to determine whether lifelong lowering of LRRK2 protein levels through LoF results in an apparent reduction in lifespan. We found no significant difference between the age distribution of *LRRK2* pLoF variant carriers and noncarriers in either the gnomAD or 23andMe datasets (two-sided Kolmogorov–Smirnov *P* = 0.085 and 0.46 respectively; Fig. 3a), suggesting no major impact on longevity, though we note that this analysis is based on age at sample collection, which is not equivalent to longevity and at current sample sizes we are only powered to detect a strong effect (Supplementary Table 6).

For a subset of studies within gnomAD, phenotype data are available from study or national biobank questionnaires or from linked electronic health records (Methods). We manually reviewed these records for all 60 of the 255 gnomAD *LRRK2* pLoF carriers with available data and recorded any phenotypes affecting the lung, liver, kidney, cardiovascular system, nervous system, immunity and cancer (Supplementary Table 7). We found no over-representation of any phenotype or phenotype category in *LRRK2* pLoF carriers.

The 23andMe dataset includes self-reported data for thousands of phenotypes across a diverse range of categories. We performed a phenome-wide association study comparing *LRRK2* pLoF carriers to noncarriers for 366 health-related traits and found no significant association between any individual phenotype and carrier status (Fig. 3b). In particular, we found no significant associations with any lung, liver or kidney phenotypes (Supplementary Tables 5 and 8).

**Fig. 4:**
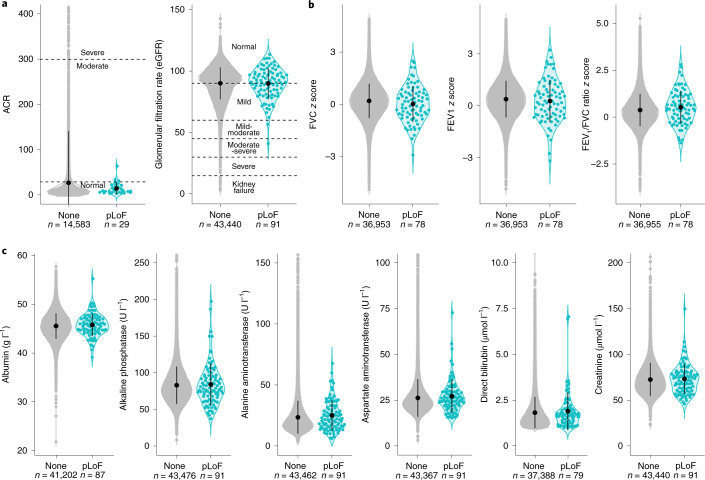
*LRRK2* pLoF carriers do not have impaired lung, liver or kidney function. For all plots, points for individual pLoF carriers are shown in teal and noncarriers in gray. The mean and 1 × s.d. are represented by the black circle and line. **a**, Urine biomarkers albumin and creatinine were transformed into two clinical markers of kidney function (Methods). No pLoF carriers showed signs of severely impaired kidney function. ACR, albumin to creatinine ratio. **b**, *Z* scores of age-, sex- and height-corrected spirometry measures of lung function^36^. FVC, forced vital capacity; FEV_1_, forced expiratory volume in 1 s. **c**, Blood serum biomarkers of liver function. The plots for alkaline phosphatase, alanine aminotransferase, aspartate aminotransferase, bilirubin and creatinine were top-truncated, removing 47, 29, 92, 8 and 27 noncarriers respectively. The violin plots and summary statistics were calculated on the full dataset. All pLoF carriers are within each plot area.

The UK Biobank resource includes measurements for 30 blood serum and four urine biomarkers. We found no difference in any of these biomarkers between pLoF carriers and noncarriers (Supplementary Table 9 and Supplementary Fig. 1). In particular, there was no difference between carriers and noncarriers for urine biomarkers transformed into clinical measures of kidney function (Fig. 4a and Methods) and no difference in six blood biomarkers commonly used to assess liver function (Fig. 4c). We also observed no difference in spirometry measurements of lung function (Fig. 4b).

We grouped self-reported disease diagnoses in UK Biobank individuals into categories corresponding to the organ system and/or mechanism (Supplementary Table 10). We observed no enrichment for any of these phenotype groups in *LRRK2* pLoF carriers when compared to noncarriers (Supplementary Table 11). We also mined ICD10 codes from hospital admissions and death records for any episodes relating to lung, liver and kidney phenotypes, removing any with a likely infectious or other external cause (Supplementary Table 12 and Methods) and identified six pLoF carriers with ICD10 codes relating to these organ systems (6.19%), compared to 4,536 noncarriers (9.87%; Supplementary Tables 13 and 14).

Our results indicate that approximately 1 in every 500 humans is heterozygous for a pLoF variant in *LRRK2*, resulting in a systemic lifelong decrease in LRRK2 protein levels and that this partial inhibition has no discernible effect on survival or health at current sample sizes. These results suggest that partial reduction of LRRK2 protein in humans is unlikely to result in the severe phenotypes observed in knockout animals. This is consistent with initial phase 1 studies of therapeutic LRRK2 kinase inhibitors, which have shown promising safety results^24^, but are not yet able to address long-term, on-target pharmacology-related safety profiles.

The rarity of pLoF variants in *LRRK2*, combined with the relatively low prevalence of PD, prevents direct assessment of whether LRRK2 inhibition reduces the incidence of PD with current sample sizes (Supplementary Table 5). Future cohorts with many more sequenced and phenotyped individuals (probably millions of samples) will be required to answer this question. As such, our study focuses entirely on whether partial genetic LRRK2 inactivation has broader phenotypic consequences that might correspond to adverse effects of chronic administration of LRRK2 inhibitors.

We acknowledge multiple limitations to this work. First, we relied on heterogeneous phenotype data mostly derived from self-reported questionnaires. Both 23andMe and gnomAD record only age at recruitment, which is an imperfect proxy for lifespan and participants are relatively young compared to the typical age of onset for PD. In addition, at current sample sizes we are only powered to detect a strong effect on lifespan. Our ascertainment of *LRRK2* pLoF variants in 23andMe was necessarily incomplete, due to the availability of targeted genotyping rather than sequencing data; this means that a subset of the 23andMe individuals treated as noncarriers could be carriers of *LRRK2* pLoF variants not genotyped or imputed in this dataset. We have not directly assessed whether *LRRK2* pLoF variants reduce kinase activity and instead take reduction in protein levels as a proxy. Previous studies have, however, shown that Rab10 phosphorylation is markedly reduced when LRRK2 levels are lowered by ~80% using siRNA^34,35^. Additionally, lifelong LoF of *LRRK2* may not be equivalent to therapeutic inactivation later in life if biological compensation occurs. Finally, the low-frequency of naturally occurring *LRRK2* pLoF variants results in a relatively small number of carriers that could be assessed for each biomarker and phenotype, meaning that we are not well powered to detect subtle or infrequent clinical phenotypes arising from LRRK2 haploinsufficiency. However, our study suggests that any clinical phenotype associated with partial reduction of LRRK2 is likely to be substantially more benign than early-onset PD.

This study provides an important proof of principle for the value of very large genetically and phenotypically characterized cohorts, combined with thorough variant curation, in exploring the safety profile of candidate drug targets. Over the coming years, the availability of complete exome or genome sequence data for hundreds of thousands of individuals who are deeply phenotyped and/or available for genotype-based recontact studies, combined with deep curation and experimental validation of candidate pLoF variants, will provide powerful resources for therapeutic target validation as well as broader studies of the biology of human genes.

## Methods

### gnomAD variant annotation and curation

The gnomAD resource, including both sample and variant quality control (including sample ancestry assignment), is fully described in our companion paper^9^. Analysis was conducted using gnomAD v.2.1.1. Putative LoF variants were defined as stop-gained, frameshift or essential splice site (splice donor or splice acceptor) as annotated by the Ensembl Variant Effect Predictor^37^.

Variants were included if they were annotated as LoF on any of the three high-confidence GENCODE annotated protein-coding transcripts that are expressed in the lung, liver or kidney. All variants also underwent transcript expression-aware annotation which evaluates cumulative expression status of transcripts harboring a variant in the Genotype Tissue Expression (GTEx) project dataset^38^. All high-confidence variants were found in exons with high evidence of expression across all relevant tissues in GTEx. In addition, all were high-confidence pLoF on the canonical transcript, which is the only transcript to include the kinase domain.

Variants were filtered out if they were flagged as low confidence by LOFTEE^9^. For the remaining variants, manual curation was performed, including inspection of variant quality metrics, read distribution and the presence of nearby variants using the integrative genome viewer and splice-site prediction algorithms using Alamut.

**Extended Data Fig. 3:**
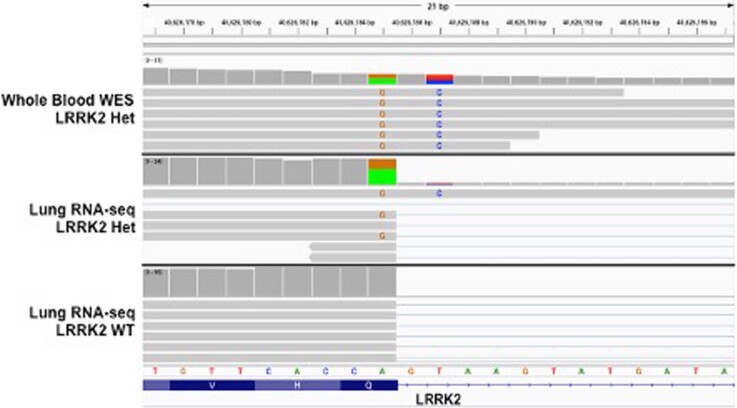
IGV visualization of the splice donor variant GRCh37:12-40626187-T-C in the GTEx *LRRK2* pLoF carrier exome sequencing data and lung tissue RNA-seq data compared to a control GTEx lung RNA-seq sample. The pLoF variant is observed on reads containing an anchoring missense variant, GRCh37:12-40626185-A-G (A green and G orange), and these reads are presenting normal splicing as seen in the control RNA-seq sample.

A single splice-site variant (12-40626187-T-C), found in 77 gnomAD carriers, was identified in an individual with RNA-seq data in the GTEx project. The RNA-seq reads were manually inspected to look for any effect on splicing. Assessing the read distribution of a linked heterozygous variant in this individual showed convincingly that the variant has no discernible effect on transcript splicing (Extended Data Fig. 3). All available tissues were assessed with reads from lung tissue shown in Extended Data Fig. 3. The variant was also identified in eight UK Biobank carriers and in 23andMe and was similarly excluded from these cohorts.

This study complied with all relevant ethical regulations and was overseen by the Broad Institute’s Office of Research Subject Protection and the Partners Human Research Committee. Informed consent was obtained from all participants.

### Sanger validation of gnomAD variant carriers

Sanger validation was performed on genomic DNA derived from peripheral blood under the following PCR conditions: 98 °C 2 min; 30 cycles 20 s 98 °C, 20 s 54 °C, 1 min 72 °C; 3 min 72 °C using Herculase II Fusion DNA polymerase (Agilent, 600679). PCR products (5 μl) were analyzed on a 2% agarose gel and the remaining product was purified with the Qiagen PCR Purification kit. Sequence analysis was performed with both PCR primers at Quintarabio. Details of variants and PCR primers used for each are listed in Supplementary Table 3.

### gnomAD phenotype curation and cohort descriptions

The below described studies with *LRRK2* pLoF carriers had available phenotype data. For each study, all available records were manually reviewed to identify any reports of health problems, which were categorized into the following classes: lung, liver, kidney, cardiovascular, nervous system, immune and cancer.

#### The genomic psychiatry cohort project

The genomic psychiatry cohort project is a longitudinal resource with the aim of making population-based data available through the National Institute of Mental Health. The repository contains whole-genome sequencing (WGS) data and detailed clinical and demographic data, particularly focused on schizophrenia and bipolar disorders. A large proportion of participants (88%) have consented for recontact^39^. The screening questionnaire consisted of 32 yes/no questions about mental health issues and 23 yes/no questions covering other medical problems including liver, digestive and cardiovascular problems. There were no specific questions relating to lung or kidney phenotypes, although participants were asked to answer yes/no to having any additional health problems. If a participant answered yes to this question, we marked the existence of lung or kidney disease as ‘unknown’. One sample was excluded due to conflicting questionnaire answers.

The age of the 25 *LRRK2* carriers ranged from 19 to 67 years. Two carriers, aged 55 and 60 years, reported having had liver problems and four participants over 60 years reported no liver problems.

#### The Pakistan risk of myocardial infarction study

The Pakistan risk of myocardial infarction study comprises 10,503 individuals characterized using a phenotype questionnaire with >350 items covering demographic and dietary characteristics and over 80 blood biomarker measurements^40^. The predominant focus of the questionnaire was cardiac function and phenotype. While the participants were specifically asked to report suffering from asthma, no other lung, liver, kidney, nervous system or immune phenotypes were directly assayed and so these were marked as ‘unknown’ for these individuals. The 12 *LRRK2* LoF carriers in the study did not differ in terms of age, sex and myocardial infarction status when compared to the entire cohort.

#### The Swedish schizophrenia and bipolar studies

Cases with schizophrenia or bipolar disorder were identified from Swedish national hospitalization registers^41,42^. Controls were selected at random from population registers. All individuals had whole-exome sequencing data^43^. All available ICD codes from inpatient hospitalizations and outpatient specialist treatment contacts were provided for each patient.

#### The national FINRISK study

The FINRISK study has been carried out for 40 years since 1972 every 5 years using independent, random and representative population samples from different parts of Finland. For this work, we used sequencing and health register data from FINRISK surveys between 1992 and 2007 (ref. ^44^).

Full health records including ICD10 codes were reviewed by study coordinators who provided us with yes/no answers for each of our phenotype classes.

#### The BioMe biobank at the Charles Bronfman Institute for Personalized Medicine at Mount Sinai

The Mount Sinai BioMe Biobank, founded in September 2007, is an ongoing, broadly consented electronic health record (EHR)-linked bio and data repository that enrolls participants nonselectively from the Mount Sinai Medical Center patient population (New York City). BioMe participants represent broad racial, ethnic and socioeconomic diversity with a distinct and population-specific disease burden, characteristic of the communities served by Mount Sinai Hospital. Currently comprising over 47,000 participants, BioMe participants are of African (24%), Hispanic/Latino (35%), European (32% of whom 40% are Ashkenazi Jewish) and other/mixed ancestry.

BioMe is linked to Mount Sinai’s system-wide Epic EHR, which captures a full spectrum of biomedical phenotypes, including clinical outcomes, covariate and exposure data from past, present and future healthcare encounters. The median number of outpatient encounters is 21 per participant, reflecting predominant enrollment of participants with common chronic conditions from primary care facilities. Clinical phenotype data have been meticulously harmonized and validated.

Genome-wide genotype data and whole-exome sequencing data are available for >30,000 participants. In addition, WGS data are available for >11,000 participants. The full EHRs of three BioMe *LRRK2* pLoF carriers were reviewed by local clinicians and we were provided with detailed summaries.

#### Estonian Biobank of the Estonian Genome Center, University of Tartu

The Estonian Biobank cohort is composed of volunteers from the general Estonian resident adult population^45^. The current number of participants of close to 165,000 (representing 15% of the Estonian adult population) makes it ideally suited to population-based studies. Participants were recruited throughout Estonia by medical personnel and participants receive a standardized health examination, donate blood and fill out a 16-module questionnaire on health-related topics such as lifestyle, diet and clinical diagnoses. A detailed phenotype summary from a health survey and linked data including ICD10 codes, clinical laboratory values and treatment and medication information is annually updated through linkage with national electronic health databases and registries.

### UK Biobank variant detection and curation

The 49,960 exome-sequenced individuals from the UK Biobank were restricted to a subset of 46,062 unrelated individuals of European ancestry. Relatedness was determined using KING kinship coefficient estimates from the genotype relatedness file with a cutoff of 0.0884 to include pairs of individuals with greater than third-degree relatedness. European ancestry was determined by projecting individuals onto the 1000 Genomes Project phase 3 (ref. ^46^) principal-component analysis (PCA) coordinate space, followed by Aberrant R package^47^ clustering to retain only those individuals falling within the 1000 Genomes Project EUR PC1 and PC2 limits (*λ* = 4.5). We further removed individuals who self-reported as non-European ethnicity.

We identified all individuals with putative LoF variants detected in the FE analysis pipeline, which used GATK 3.0 for variant calling and filtering^33^. We did not use the SPB pipeline calls due to advertised errors in the Regeneron Genetics Center pipeline at the time we were conducting these analyses. Variants were included if they were annotated as LoF on any transcript expressed in the lung, liver or kidney. As with the gnomAD analysis, variants were filtered out if they were flagged as low confidence by LOFTEE, before manual curation of the remaining variants. This curation included inspection of variant quality metrics, read distribution and the presence of nearby variants using integrative genome viewer and splice-site prediction algorithms using Alamut.

In addition, 266 individuals in the full genotyped cohort of 488,288 samples who were carriers of the G2019S risk allele were identified. One individual who was a carrier for both a *LRRK2* pLoF variant and G2019S was excluded from all analyses. Carriers of G2019S were not included in the ‘noncarrier’ cohort in any of the analyses.

**Extended Data Fig. 4:**
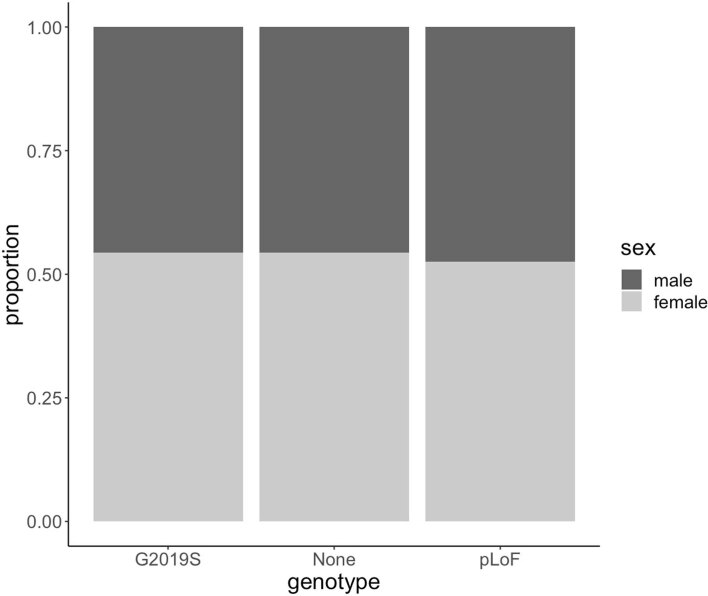
Sex distribution of *LRRK2* pLoF carriers, G2019S risk allele carriers and non-carriers in the UK Biobank. Males are shown in dark grey and females in light grey.

**Extended Data Fig. 5:**
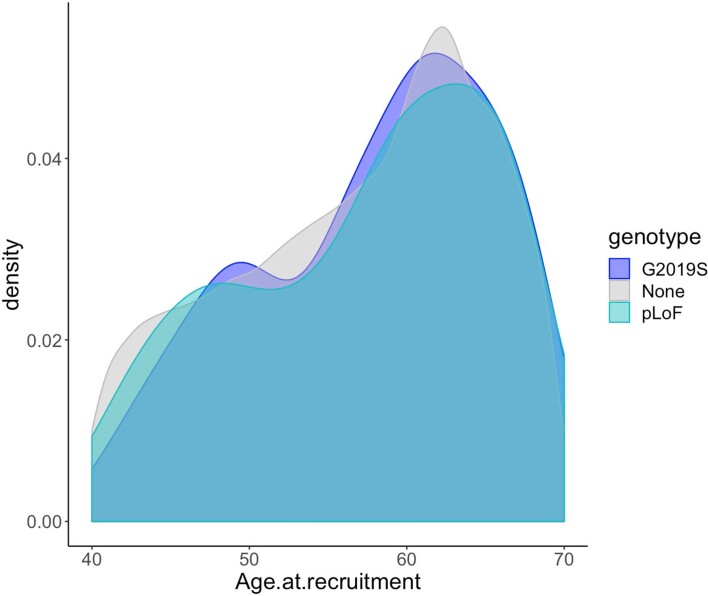
Age distribution of *LRRK2* pLoF carriers, G2019S risk allele carriers and non-carriers in the UK Biobank. Data are shown as overlapping density plots.

*LRRK2* pLoF carriers, G2019S risk allele carriers and noncarriers are well matched for both sex (Extended Data Fig. 4) and age (Extended Data Fig. 5).

### UK Biobank phenotype analysis

#### Blood serum and urine biomarkers

The first recorded value of all fields relating to ‘blood biochemistry’ (field codes 30600–30890) and ‘urine assays’ (field codes 30510–30535) was extracted for all individuals. The distribution of values for all biomarkers was plotted (Supplementary Fig. 1) and a two-sided Wilcoxon test was used to test for a difference between *LRRK2* pLoF carriers and noncarriers.

These data were also extracted for G2019S risk allele carriers and these individuals were compared to both pLoF carriers and carriers of neither G2019S nor *LRRK2* pLoF variants. There was no significant difference in any of the 34 biomarkers between pLoF and G2019S carriers after accounting for multiple testing (Supplementary Table 15). When comparing G2019S carriers to noncarriers we found significant associations with cystatin C and phosphate levels.

#### Clinical measures of kidney function

ACR was calculated by dividing the urine microalbumin value (field code 30500; mg l^−1^) by the urine creatinine value (field code 30510; μmol l^−1^) multiplied by a factor of 0.0001131222. Estimated glomerular filtration rate was calculated using the CKD Epidemiology Collaboration (CKD-EPI) creatinine equation^48^. Normal range values for both ACR and estimated glomerular filtration rate were taken from the National Kidney Foundation website (https://www.kidney.org/kidneydisease/).

#### Spirometry measures of lung function

To assess lung function we used Global Lung Initiative 2012 reference equation *z* scores standardized for age, sex and height for FEV_1_, FVC and FEV_1_/FVC ratio measured using spirometry. These calculations are available in field codes 20256, 20257 and 20258 and were described previously^36^.

#### Grouped phenotype analysis

The list of all codings within the field ‘20002 Non-cancer illness code, self-reported’, were taken from the UK Biobank showcase (http://biobank.ctsu.ox.ac.uk/crystal/coding.cgi?id=6). All selectable codings were given a primary grouping pertaining to the main system relating to that disease. In rare instances where more than one grouping could be assigned, the second was included as a secondary grouping. Diseases with an autoimmune basis were given a secondary grouping to reflect a similar underlying mechanism. Due to the opposing effects of some respiratory diseases, where appropriate, phenotypes in this category were given a secondary grouping of airway, interstitial or pleural. Any codings reflecting symptoms, trauma/injury, benign cancer, mental health phenotypes or diseases arising as a result of infection were excluded. All phenotype codings and assigned groupings are listed in Supplementary Table 10. Any coding within the field ‘20001 Cancer code, self-reported’ was assigned a grouping of ‘cancer’.

To test for an association between any phenotype group and *LRRK2* pLoF carrier status, each individual was counted once as either having self-reported any of the phenotypes within a group or having reported none. A Fisher’s exact test was used to test for an association.

#### Analysis of ICD10 codes

All ICD10 codes relating to diseases of the liver (K70–K77), diseases of the respiratory system not specific to the upper respiratory tract (J20–J22, J40–J47, J80–J99) or kidney diseases (N00–N29) were curated to exclude any with a primary infectious or external cause (Supplementary Table 12). Asthma was excluded from all analyses to avoid any issues caused by the deliberate ascertainment of the exome-sequenced portion of the cohort on the basis of asthma status.

For each individual, we extracted all ICD10 codes from the fields ‘41270 Diagnoses: ICD10’ (recorded from episodes in hospital), ‘40001 Underlying (primary) cause of death: ICD10’ and ‘40002 Contributory (secondary) causes of death: ICD10’. The number of carriers and noncarriers with any ICD10 code relating to lung (5 pLoF carriers; 2,378 noncarriers), liver (0 pLoF carriers; 652 noncarriers) or kidney disease (3 pLoF carriers; 2,272 noncarriers) were counted. For J43 (emphysema), J44 (other chronic obstructive pulmonary disease) and J47 (bronchiectasis), ICD10 codes were not counted if they were reported alongside exposure to or history of tobacco use (Z77.22, P96.81, Z87.891, Z57.31, F17 or Z72.0).

### 23andMe variant annotation and curation

23andMe participants have been genotyped on a variety of platforms and imputed against a reference panel comprising 56.5 million variants from the 1000 Genomes Project phase 3 (ref. ^46^) and UK10K^49^. Putative *LRRK2* LoF variants were defined as those classified as high confidence by LOFTEE. Variants were manually assessed for call rate, genotyping and imputation quality and manually curated to ensure they were expected to cause true LoF.

For each of the two genotyped *LRRK2* pLoF, we determined carrier status by manually inspecting and custom calling the probe intensity plots. For the imputed variants, carrier status was determined from the minimac-imputed dosage. As these calling methods might produce false positives, we confirmed the participants’ genotypes through Sanger sequencing. Individuals with discordant genotypes were excluded. This resulted in a cohort of 749 individuals, each of whom is a Sanger sequence-confirmed carrier for one of three pLoF variants (Supplementary Table 4).

During initial selection and sequencing, expansion of the database led to inclusion of a number of additional individuals genotyped for one of the pLoF variants, rs183902574. We performed custom calling on these individuals and found 354 deemed as high-confidence carriers (Supplementary Table 4). As these individuals were not Sanger sequenced, all subsequent analyses were performed both including and excluding these individuals.

Participants provided informed consent and participated in the research online, under a protocol approved by the institutional review board, Ethical & Independent Review Services, an organization accredited by the Association for the Accreditation of Human Research Protection Programs.

### Testing the power to detect an age effect in 23andMe

As a positive control for age analysis, we tested the apolipoprotein E (APOE) Alzheimer’s disease risk allele rs429358, which has a known effect on lifespan. This effect is highly significant in this dataset (*P* = 1.2 × 10^−^^211^).

Given that the carrier count for rs429358 is much higher than for *LRRK2* pLoF, we assessed the power of the 23andMe dataset to detect an age effect associated with LRRK2 pLoF variants that is of the same effect size as the known effect of the APOE allele rs429358 by sampling carriers of this variant. We randomly selected *N* carriers of rs429358 from the 23andMe dataset, performed a Kolmogorov–Smirnov test on the age distribution of those carriers versus 4,000,000 noncarriers and considered the resulting *P* value. We repeated this process 100 times and then computed the proportion of these simulations with *P* < 0.05. This tells us our power to reject the null hypothesis that APOE does not have an effect on age at *α* = 0.05, if we had *N* carriers in the dataset. We repeated this for different values of *N* between 1,000 and 20,000 (Supplementary Table 6).

### Association testing in the 23andMe dataset

#### Phenotype selection

The 23andMe dataset includes self-reported phenotype data for thousands of phenotypes across a diverse range of categories. These phenotypes have different sample sizes and prevalence, so the power to detect associations varies widely. We began with a curated set of 748 disease phenotypes. We then applied a liberal filter based on our power to detect an association with carrier status. More specifically, assuming a minor allele frequency of 2 × 10^−5^, we restricted to phenotypes where we had power 0.1 to detect an association effect with odds ratio (OR) > 1.3 (for binary traits) or *β* > 0.2 (for quantitative traits) at *α* = 0.0001 significance. This left us with 460 binary and 14 quantitative phenotypes.

#### Association testing

For the subset of 366 health-related phenotypes (excluding any related to diet, drug use, lifestyle and personality), we first restricted testing to individuals for whom we had phenotypic data. We calculated pairwise identity by descent (IBD) over all individuals using a modified version of the IBD64 program and then iteratively removed individuals until we were left with a set of participants, no two of whom shared >700 cM in IBD. We then tested the association between phenotype and carrier status, controlling for age, sex, genotyping platform and the first ten genetic principle components. We used logistic regression for binary phenotypes and linear regression for quantitative phenotypes.

To control for population structure we restricted our analyses to participants with >97% European ancestry, but the results did not qualitatively change when we dropped this restriction. We also tested associations using only individuals whose carrier status was confirmed by Sanger sequencing, but this also did not result in any meaningful difference.

A Bonferroni-corrected *P* value threshold for 366 independent tests of 1.37 × 10^−4^ was used to assess statistical significance.

#### Power analysis

For each phenotype, we computed the theoretical OR we were powered to detect (given in Supplementary Tables 5 and 8) as follows: let *m* be the proportion of individuals used in the association study of that phenotype who are LRRK2 pLoF carriers and let *n*0 and *n*1 be the number of controls and cases, respectively. For each OR in the interval (1, 10) at steps of 0.02, we computed the power of the Cochran–Armitage trend test to detect an association between a variant with minor allele frequency *m* and OR at *α* = 0.05, with *n*0 controls and *n*1 cases^50^. We reported the smallest OR such that the power was ≥0.8.

### Analysis of LRRK2 protein levels

#### Cell culture

All cell lines tested negative for *Mycoplasma* contamination on a monthly basis with the MycoAlert Detection kit (Lonza, LT07-118) and MycoAlert Assay Control Set (Lonza, LT07-518). Cells were grown at 37 °C with 5% CO_2_.

#### Human embryonic stem cell culture

All pluripotent stem cells were approved by Harvard ESCRO protocol E00052 and E00067. Human ESCs (hESCs) were obtained from WiCell Research Institute (WA01, H1) under a material transfer agreement. Cell lines were authenticated by visual inspection of cell morphology with bright-field microscopy, staining with anti-Oct4 antibody to determine maintenance of pluripotency (Santa Cruz, sc-5279, data not shown), sent to WiCell Research Institute after 6 months of passaging or after isogenic cell line generation for karyotyping and in some cases PCA of RNA-seq data to confirm clustering with other pluripotent stem cell lines. Pluripotent stem cells were plated onto hESC-qualified Matrigel (VWR, BD354277)-coated six-well plates, mTeSR1 medium was changed daily (StemCell Technologies, 85850) and cells were passaged every 5–7 d with 0.5 mM EDTA.

#### Lymphoblastoid cell culture

LCLs were obtained from Coriell Biorepository (GM18500, GM18501, GM18502, HG01345, HG01346) and approved by the Broad Institute Office of Research Subject Protection protocol 3639. Cell lines were authenticated by visual inspection of cell morphology with bright-field microscopy and in some cases PCA of RNA-seq data to confirm clustering with GTEx LCLs. LCL medium was changed every other day with RPMI 1640 medium (Life Technologies), 2 mM l-glutamine (Life Technologies) and 15% FBS (Sigma).

#### Cardiomyocyte differentiation

Cardiomyocyte differentiation of the control and engineered H1 hESC lines was performed according to the protocol by Lian et al.^51^. Briefly, 500,000 cells were plated on hESC-qualified Matrigel (VWR, BD354277), grown in mTeSR1 medium for 4 d (StemCell Technologies, 85850) and switched to RPMI medium (Life Technologies) with B27 supplement (Life Technologies), switching to B27 with insulin at day 7 for the remainder of the protocol. On day 0 of differentiation, 12 µM CHIR99021 (Tocris) was applied for 24 h. At day 3, cultures were treated with 5 µM IWP2 (Tocris) for 24 h. Bright-field images and movies were acquired at day 17 and cells were collected for protein/RNA extraction at day 19.

#### Isogenic cell line engineering

The following guide and homologous recombination (HR) template were delivered into single cell H1 hESCs via nucleofection (Lonza 4D-Nucleofector X unit) using the P3 Primary Cell kit (V4XP-3024), pulse code CA137 and pX459 (Addgene): AATAAGGCATTTCATATAGT and ACAGGCCTGTGATAGAGCTTCCCCATTGT GAGAACTCTGAAATTATCATCTGACTATATGAAATGCCTTATTTTCCAATGGGATTTTGGTCAAGATTAA. Cells were allowed to recover from nucleofection in mTeSR supplemented with 10 µM Rock Inhibitor (Y-27632, Tocris) overnight. For the following 3 d the cells were treated with 0.25 µg ml^−1^ puromycin (VWR) in mTeSR. Cells were then cultured in mTeSR until colonies were ready to be split. Engineered cells were split into single cells and plated in Matrigel-coated 96-well plates at a density of 0.5 cells per well. Plates were screened for colonies 8–10 d after plating and grown until colonies were ready to be split. Colonies were then split with 0.5 mM EDTA into two identical 96-well plates, one for DNA extraction/PCR/sequencing and one for freezing cells. Once colonies were ready to be split, 96-well plates were frozen in mFreSR (Stem Cell Technologies) and stored at −80 °C until HR-positive wells were identified. HR-edited cells were then thawed and expanded for four generations, validated by Sanger sequencing, karyotyping and OCT4 staining before proceeding with cardiomyocyte differentiation.

#### Off-target analysis of CRISPR/Cas9 engineering

To detect any potential off-target effects caused by CRISPR/Cas9 genome editing, WGS was conducted for both engineered and control cell lines. DNA extraction, quality control and 30× PCR-free WGS were performed by the Genomics Platform at the Broad Institute. An AllPrep DNA/RNA extraction kit was used, following its protocol. Alignment, marking of duplicates, recalibration of quality scores and variant calling were all performed using GATK best practices^52^.

We identified 157,230 variants in the engineered cell line that were not found in the control cell line as candidate variants. For the guide RNA (gRNA) used, we defined potential off-target regions as those with a <4-bp mismatch against the full 20-bp gRNA sequence (334 regions) and/or a <2-bp mismatch against the seed (PAM proximal) 12 bp of the gRNA sequence (5,780 regions), each followed by the NGG PAM. We looked for any candidate variant that fell into the potential off-target region, resulting in detection of only one variant (chr8-65084564-A-AT) that fell onto a region with one mismatch against the seed 12 bp of gRNA sequence (chr8:65084560-65084575). No variants with a <4-bp mismatch against the full 20-bp gRNA sequence or perfect match at the seed region were detected. Because a mismatch at the seed region decreases the likelihood of off-target variants and also because the single variant we detected is a known variant (rs1161563412) observed in the population without apparent phenotypic association, we concluded that no major off-target effect exists at the level of violating the main steps of our research. All the analysis for the detection of potential off-targets were conducted using pybedtools^53^ and CRISPRdirect^54^ software.

#### Western blot analysis

Cell pellets were snap-frozen in liquid nitrogen and stored at −80 °C. Cells were Dounce-homogenized in ice-cold radioimmunoprecipitation assay buffer (89901; Thermo Fisher Scientific) containing protease inhibitors (Halt Protease Inhibitor, Thermo Fisher Scientific). Homogenates were rotated at 4 °C for 30 min, followed by centrifugation at 15,000*g* for 20 min at 4 °C. Equal amounts of protein (50 µg) were electrophoresed on 4–20% SDS–PAGE (Bio-Rad) and transferred to nitrocellulose membranes. The following antibodies were used for immunoblotting: LRRK2 (75-253, UC Davis/National Institutes of Health NeuroMab Facility), anti-actinin (A7811, Sigma), GAPDH (sc-25778, Santa Cruz), anti-rabbit IgG HRP (7074, Cell Signaling) and anti-mouse IgG HRP (7076, Cell Signaling). Immunoblots were developed using enhanced chemiluminescence (SuperSignal West Pico Chemiluminescent Substrate, Thermo Fisher Scientific) on an Amersham Imager 600.

### Reporting Summary

Further information on research design is available in the Nature Research Reporting Summary linked to this article.

## Online content

Any methods, additional references, Nature Research reporting summaries, source data, extended data, supplementary information, acknowledgements, peer review information; details of author contributions and competing interests; and statements of data and code availability are available at 10.1038/s41591-020-0893-5.

## Supplementary information

Supplementary Information Supplementary Fig. 1.

Reporting Summary

Supplementary Tables Supplementary Tables 1–15.

## Data Availability

The gnomAD 2.1.1 dataset is available for download at http://gnomad.broadinstitute.org, where we have developed a browser for the dataset and provide files with detailed frequency and annotation information for each variant. There are no restrictions on the aggregate data released. The UK biobank resource was accessed under application number 42890.

## Source data

Source Data Fig. 2 Unprocessed immunoblots for Fig. 2.

## Code availability

The code used to make the figures is available at https://github.com/macarthur-lab/gnomad_lrrk2.
