# Supplementary material for: The effect of LRRK2 loss-of-function variants in humans
Source: Nat Med. 2020 May 27;26(6):869–77. doi: 10.1038/s41591-020-0893-5 (PMC7303015; doi:10.1038/s41591-020-0893-5)
Supplement: Supplementary file 1 — Supplementary Fig. 1. [file 41591_2020_893_MOESM1_ESM.pdf]

natureresearch
https://doi.org/10.1038/s41591-020-0893-5

Supplementary information

The effect of LRRK2 loss-of-function variants in humans

In the format provided by the authors and unedited

Supplementary Figure 1: Comparison of 30 blood serum and 4 urine biomarkers between *LRRK2* pLoF carriers (teal), G2019S risk allele carriers (blue) and carriers of neither (None; grey). The mean and standard deviation in each cohort are shown by black circles and lines respectively. For some biomarkers plots have been top truncated to remove outlines in the non-carrier cohort. Values for all pLoF and G2019S carriers are shown within each plot area. In each case, summary statistics are calculated on the full dataset.

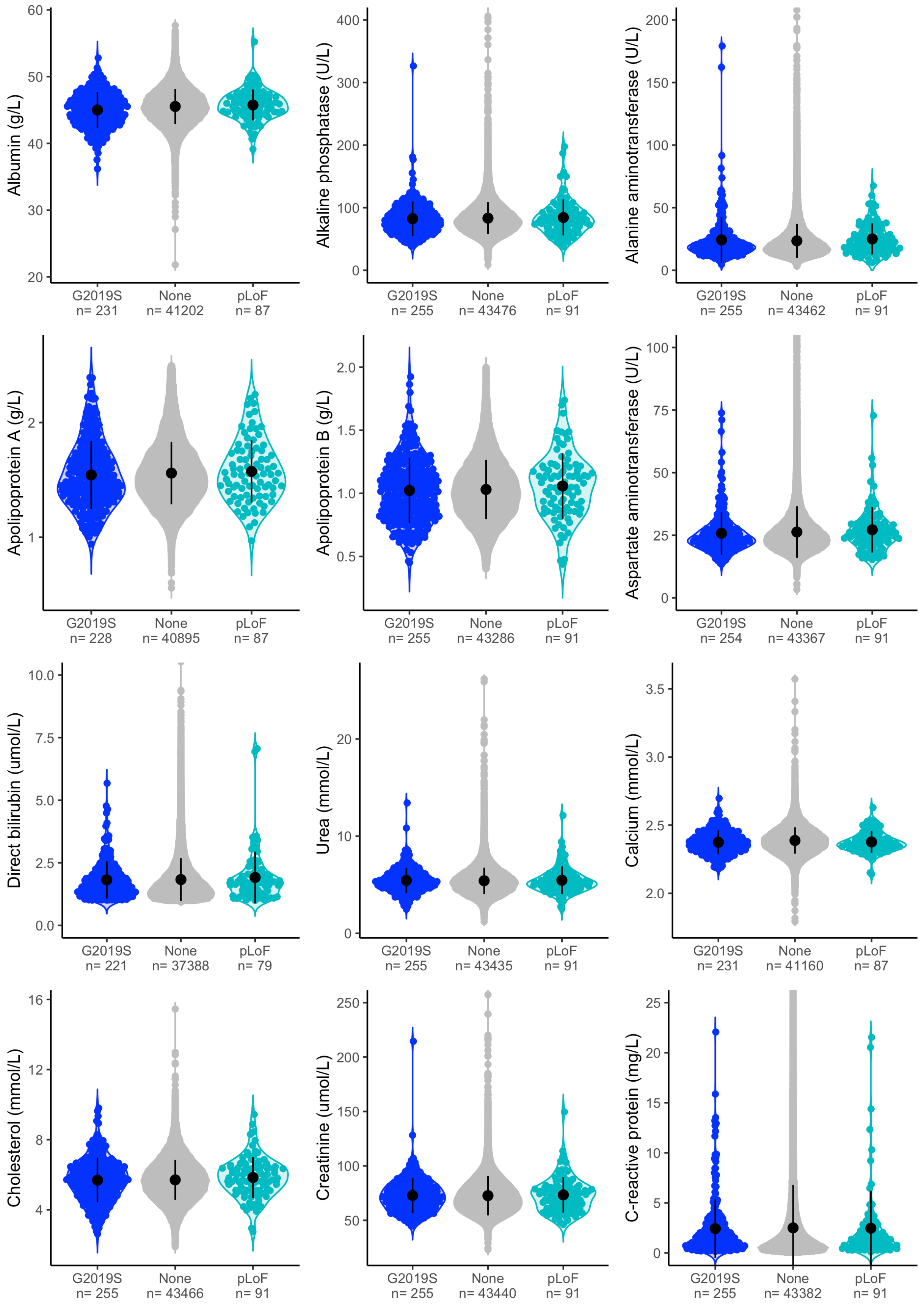
Albumin (g/L)
G2019S n= 231
None n= 41202
pLoF n= 87
Alkaline phosphatase (U/L)
G2019S n= 255
None n= 43476
pLoF n= 91
Alanine aminotransferase (U/L)
G2019S n= 255
None n= 43462
pLoF n= 91
Apolipoprotein A (g/L)
G2019S n= 228
None n= 40895
pLoF n= 87
Apolipoprotein B (g/L)
G2019S n= 255
None n= 43286
pLoF n= 91
Aspartate aminotransferase (U/L)
G2019S n= 254
None n= 43367
pLoF n= 91
Direct bilirubin (umol/L)
G2019S n= 221
None n= 37388
pLoF n= 79
Urea (mmol/L)
G2019S n= 255
None n= 43435
pLoF n= 91
Calcium (mmol/L)
G2019S n= 231
None n= 41160
pLoF n= 87
Cholesterol (mmol/L)
G2019S n= 255
None n= 43466
pLoF n= 91
Creatinine (umol/L)
G2019S n= 255
None n= 43440
pLoF n= 91
C-reactive protein (mg/L)
G2019S n= 255
None n= 43382
pLoF n= 91

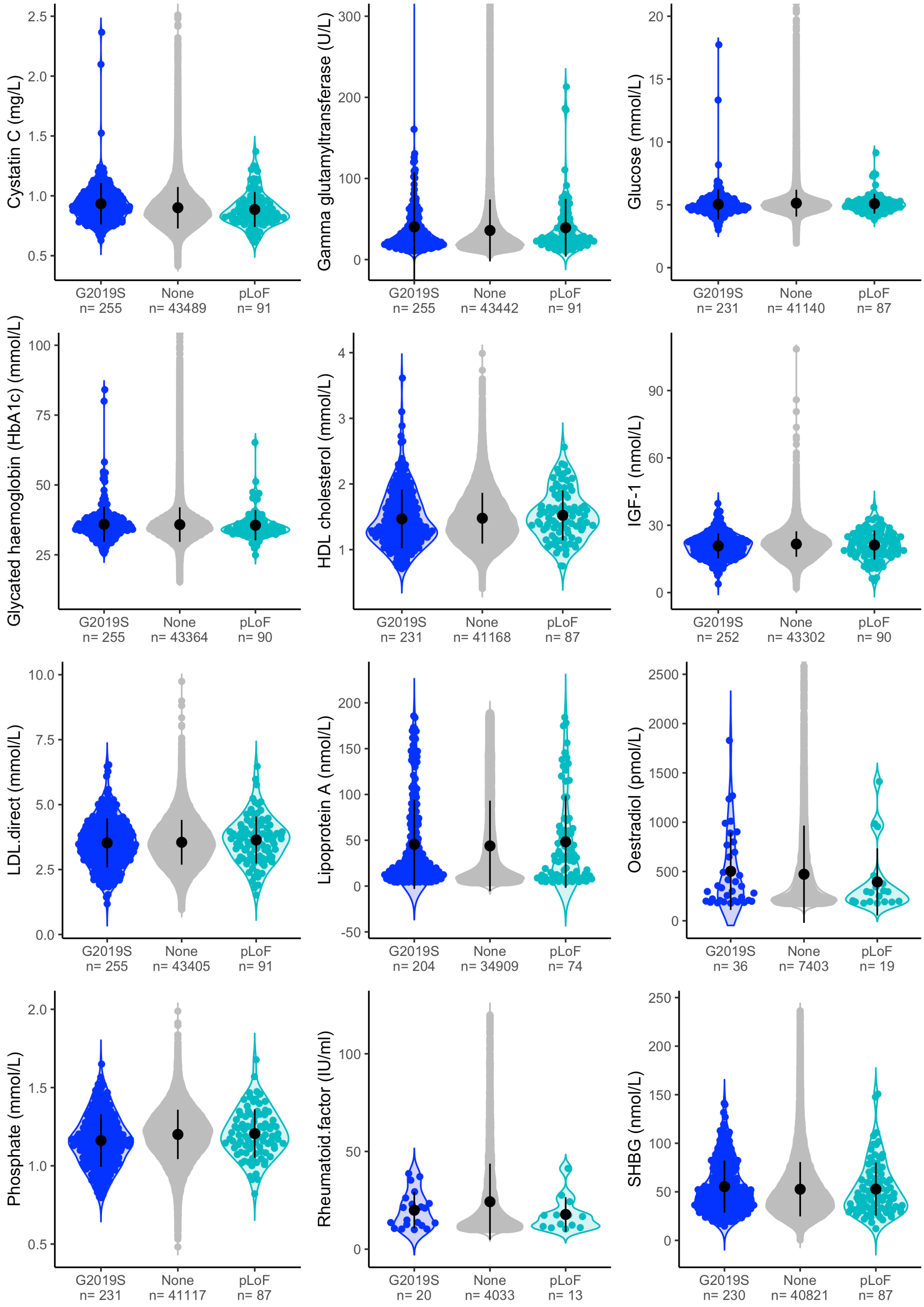
Cystatin C (mg/L)
G2019S n= 255
None n= 43489
pLoF n= 91
Gamma glutamyltransferase (U/L)
G2019S n= 255
None n= 43442
pLoF n= 91
Glucose (mmol/L)
G2019S n= 231
None n= 41140
pLoF n= 87
Glycated haemoglobin (HbA1c) (mmol/L)
G2019S n= 255
None n= 43364
pLoF n= 90
HDL cholesterol (mmol/L)
G2019S n= 231
None n= 41168
pLoF n= 87
IGF-1 (nmol/L)
G2019S n= 252
None n= 43302
pLoF n= 90
LDL.direct (mmol/L)
G2019S n= 255
None n= 43405
pLoF n= 91
Lipoprotein A (nmol/L)
G2019S n= 204
None n= 34909
pLoF n= 74
Oestradiol (pmol/L)
G2019S n= 36
None n= 7403
pLoF n= 19
Phosphate (mmol/L)
G2019S n= 231
None n= 41117
pLoF n= 87
Rheumatoid.factor (IU/ml)
G2019S n= 20
None n= 4033
pLoF n= 13
SHBG (nmol/L)
G2019S n= 230
None n= 40821
pLoF n= 87

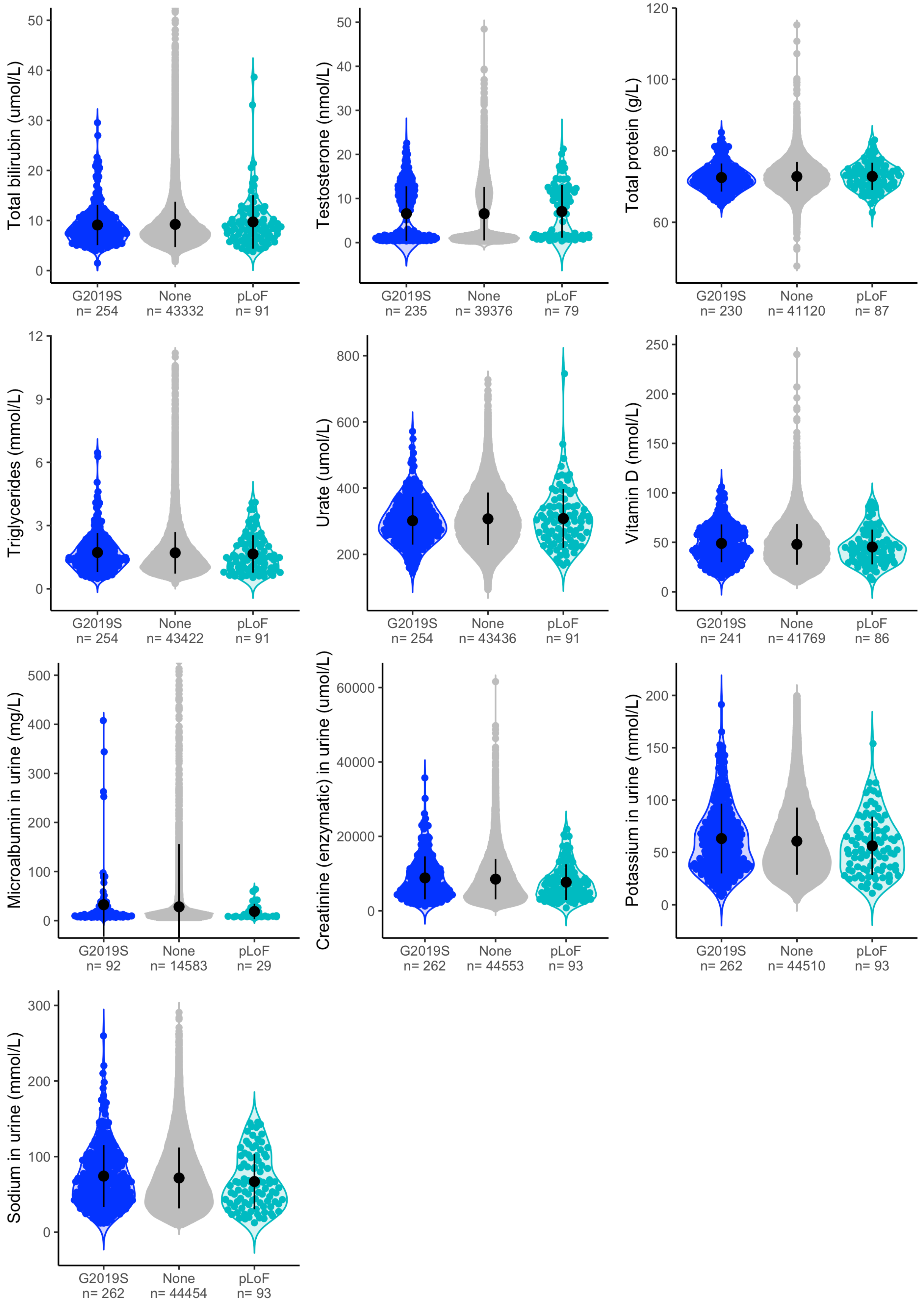

Total bilirubin (umol/L)
G2019S n= 254
None n= 43332
pLoF n= 91
Testosterone (nmol/L)
G2019S n= 235
None n= 39376
pLoF n= 79
Total protein (g/L)
G2019S n= 230
None n= 41120
pLoF n= 87
Triglycerides (mmol/L)
G2019S n= 254
None n= 43422
pLoF n= 91
Urate (umol/L)
G2019S n= 254
None n= 43436
pLoF n= 91
Vitamin D (nmol/L)
G2019S n= 241
None n= 41769
pLoF n= 86
Microalbumin in urine (mg/L)
G2019S n= 92
None n= 14583
pLoF n= 29
Creatinine (enzymatic) in urine (umol/L)
G2019S n= 262
None n= 44553
pLoF n= 93
Potassium in urine (mmol/L)
G2019S n= 262
None n= 44510
pLoF n= 93
Sodium in urine (mmol/L)
G2019S n= 262
None n= 44454
pLoF n= 93
