# Supplementary material for: The effect of LRRK2 loss-of-function variants in humans
Source: Nat Med. 2020 May 27;26(6):869–77. doi: 10.1038/s41591-020-0893-5 (PMC7303015; doi:10.1038/s41591-020-0893-5)
Supplement: Supplementary file 2 — Reporting Summary [file 41591_2020_893_MOESM2_ESM.pdf]

nature research

Corresponding author(s): Daniel G MacArthur and Nicola Whiffin

Last updated by author(s): April 14th 2020

Reporting Summary

Nature Research wishes to improve the reproducibility of the work that we publish. This form provides structure for consistency and transparency in reporting. For further information on Nature Research policies, see Authors & Referees and the Editorial Policy Checklist.

Statistics

For all statistical analyses, confirm that the following items are present in the figure legend, table legend, main text, or Methods section.

n/a	Confirmed	
☐	☒	The exact sample size (*n*) for each experimental group/condition, given as a discrete number and unit of measurement
☐	☒	A statement on whether measurements were taken from distinct samples or whether the same sample was measured repeatedly
☐	☒	The statistical test(s) used AND whether they are one- or two-sided<br>*Only common tests should be described solely by name; describe more complex techniques in the Methods section.*
☐	☒	A description of all covariates tested
☐	☒	A description of any assumptions or corrections, such as tests of normality and adjustment for multiple comparisons
☐	☒	A full description of the statistical parameters including central tendency (e.g. means) or other basic estimates (e.g. regression coefficient) AND variation (e.g. standard deviation) or associated estimates of uncertainty (e.g. confidence intervals)
☐	☒	For null hypothesis testing, the test statistic (e.g. *F*, *t*, *r*) with confidence intervals, effect sizes, degrees of freedom and *P* value noted<br>*Give P values as exact values whenever suitable.*
☒	☐	For Bayesian analysis, information on the choice of priors and Markov chain Monte Carlo settings
☒	☐	For hierarchical and complex designs, identification of the appropriate level for tests and full reporting of outcomes
☐	☒	Estimates of effect sizes (e.g. Cohen's *d*, Pearson's *r*), indicating how they were calculated

Our web collection on statistics for biologists contains articles on many of the points above.

Software and code

Policy information about availability of computer code

Data collection	No software was used to collect the data used in this study.
Data analysis	Data analysis Custom code was used to analyse data and create the figures in this study. This code has been shared in a GitHub repository. The Genome Analysis ToolKit (GATK) was used to analyses sequencing data generated from CRISPR edited cell lines, according to best practice guidelines. Pybedtools and CRISPRdirect software were used to detect potential off-target edits. The Integrative genome viewer (IGV) and Alamut software were used to curate variants.

For manuscripts utilizing custom algorithms or software that are central to the research but not yet described in published literature, software must be made available to editors/reviewers. We strongly encourage code deposition in a community repository (e.g. GitHub). See the Nature Research guidelines for submitting code & software for further information.

Data

Policy information about availability of data

All manuscripts must include a data availability statement. This statement should provide the following information, where applicable:

- Accession codes, unique identifiers, or web links for publicly available datasets
- A list of figures that have associated raw data
- A description of any restrictions on data availability

The data presented in this manuscript and the code used to make the figures are available in https://github.com/macarthur-lab/gnomad_lrrk2.

Field-specific reporting

Please select the one below that is the best fit for your research. If you are not sure, read the appropriate sections before making your selection.

☒ Life sciences ☐ Behavioural & social sciences ☐ Ecological, evolutionary & environmental sciences

For a reference copy of the document with all sections, see nature.com/documents/nr-reporting-summary-flat.pdf

Life sciences study design

All studies must disclose on these points even when the disclosure is negative.

Sample size	This study was opportunistic, and involved secondary use of all available data. No sample size was predetermined. We have included the theoretical odds ratios that our sample size is powered to detect for our pheWAS study with the 23andMe data, and a power analysis for our age distribution analysis to demonstrate the size of effect that our sample size can discover.
Data exclusions	Identified loss-of-function variant carriers were extensively curated and variants excluded if they were flagged as low-confidence by the Loss-of-function Transcript Effect Estimator (LOFTEE), or failed a manual inspection of variant quality metrics, read distribution and the presence of nearby variants that could rescue the loss-of-function effect. An additional variant was removed as it was shown to not fully disrupt splicing in the GTEx dataset. Variants from 23andMe were excluded if there were fewer than 5 Sanger validated carriers. Finally, one sample was removed from the UK Biobank that was a carrier of both a LRRK2 pLoF variant and the G2019S risk allele.
Replication	We did not attempt to reproduce any findings in a separate dataset, instead all available data was used for discovery analyses.
Randomization	As this was a population-based study, no randomization was performed.
Blinding	Blinding was not relevant for this population-based study.

Reporting for specific materials, systems and methods

We require information from authors about some types of materials, experimental systems and methods used in many studies. Here, indicate whether each material, system or method listed is relevant to your study. If you are not sure if a list item applies to your research, read the appropriate section before selecting a response.

Materials & experimental systems

n/a	Involved in the study	
☐	☒	Antibodies
☐	☒	Eukaryotic cell lines
☒	☐	Palaeontology
☒	☐	Animals and other organisms
☐	☒	Human research participants
☒	☐	Clinical data

Methods

n/a	Involved in the study	
☒	☐	ChIP-seq
☒	☐	Flow cytometry
☒	☐	MRI-based neuroimaging

Antibodies

Antibodies used

The following antibodies were used for immunoblotting: LRRK2 (1:500, 75-253, UC Davis/NIH NeuroMab Facility, lot# 455.7JD.04, clone N241A/34), α-actinin (1:5000, A7811, Sigma, Batch# 024M4758, monoclonal EA-53), GAPDH (1:10,000, sc-25778, Santa Cruz, FL-335), anti-rabbit IgG HRP (1:5000, 7074, Cell Signaling), and anti-mouse IgG HRP (1:5000, 7076, Cell Signaling).

Validation

These antibodies were selected based on their use in other publications and validation through IF and WB by the companies providing the antibodies. Company provided information is as follows:

LRRK2: Immunogen: Fusion protein amino acids 970-2527 (C-terminus) of human LRRK2 (also known as Leucine-rich repeat serine/threonine-protein kinase 2, Dardarin and PARK8, accession number Q5S007) Mouse: 89% identity (1393/1557 amino acids identical) Rat: 89% identity (1392/1557 amino acids identical) <30% identity with LRRK1 Epitope mapped to within amino acids 1836-1845 (EGDLLVNPDQ) by PEPperPRINT through work funded by The Michael J. Fox Foundation for Parkinson's Research. Monoclonal antibody info: Mouse strain: Balb/C Myeloma cell: SP2/0 Mouse Ig Isotype: IgG2a NeuroMab Applications: Immunoblot, Immunocytochemistry, Immunohistochemistry and Immunoprecipitation Species Reactivity: human, rat, mouse MW: > 200 kDa Immunoblot versus crude membranes from adult rat brain (RBM) and wild-type (WT) and LRRK2 KO mouse brains probed with N241A/34 (left) and K89/41 (right) TC supe. Mouse brain samples provided by Xiaojie Li, Ted Dawson and Valina Dawson (Johns Hopkins University). Adult rat brain immunohistochemistry (with antigen retrieval via sodium citrate pretreatment).

α-actinin: from product sheet, antibody validated for Immunoblotting (chemiluminescent) in rat leg muscle at 1:5000, 100kDa

GAPDH: GAPDH (FL-335) is recommended for detection of GAPDH and GAPDH-2 of mouse, rat, and human origin by Western Blotting (starting dilution 1:200, dilution range 1:100-1:1000), immunoprecipitation [1-2 µg per 100-500 µg of total protein (1 ml of cell lysate)], immunofluorescence (starting dilution 1:50, dilution range 1:50- 1:500) and immunohistochemistry (including paraffin-embedded sections) (starting dilution 1:50, dilution range 1:50-1:500). Molecular Weight of GAPDH: 37 kDa. Positive Controls: 293T Lysate: sc-159909, Hep G2 cell lysate: sc-2227 or KNRK whole cell lysate: sc-2214.

anti-rabbit IgG HRP: Product Description - Designed for use with rabbit polyclonal and monoclonal antibodies, this affinity purified goat anti-rabbit IgG (heavy and light chain) antibody is conjugated to horseradish peroxidase(HRP) for chemiluminescent detection. This product is thoroughly validated with CST primary antibodies and will work optimally with the CST western immunoblotting protocol, ensuring accurate and reproducible results.
Product Usage Information - Recommended Antibody Dilutions:
1:1000–1:3000
20X LumiGLO® Reagent and 20X Peroxide #7003 1:1K–1:3K
SignalFireTM ECL Reagent #6883 1:1K–1:3K
SignalFireTM Plus ECL Reagent #12630 1:5K-1:15K
SignalFireTM Elite ECL Reagent #12757 1:10K-1:20K

anti-mouse IgG HRP: Affinity purified horse anti-mouse IgG (heavy and light chain) antibody is conjugated to horseradish peroxidase(HRP) for chemiluminescent detection. This product is thoroughly validated with CST primary antibodies and will work optimally with the CST western immunoblotting protocol, ensuring accurate and reproducible results.
Product Usage Information - Recommended Antibody Dilutions:
1:1000–1:3000
20X LumiGLO® Reagent and 20X Peroxide #7003 1:1K–1:3K
SignalFireTM ECL Reagent #6883 1:1K–1:3K
SignalFireTM Plus ECL Reagent #12630 1:5K-1:15K
SignalFireTM Elite ECL Reagent #12757 1:10K-1:20K

Eukaryotic cell lines

Policy information about cell lines

Cell line source(s)	Human embryonic stem cells (hESCs) were obtained from WiCell Research Institute (WA01, H1) under an MTA. Lymphoblastoid cell lines (LCLs) were obtained from Coriell Biorepository (GM18500, GM18501, GM18502, HG01345, HG01346) and approved by the Broad Institute Office of Research Subject Protection protocol #3639.
Authentication	Cell lines were authenticated by visual inspection of cell morphology with brightfield microscopy, staining with anti-Oct4 antibody to determine maintenance of pluripotency (Santa Cruz, sc-5279, data not shown), sent to WiCell Research Institute after 6 months of passaging or after isogenic cell line generation for karyotyping, and in some cases PCA of RNA sequencing data to confirm clustering with other pluripotent stem cell lines or lymphoblastoid cell lines, as appropriate.
Mycoplasma contamination	All cell lines tested negative for mycoplasma contamination on a monthly basis with the MycoAlertTM Detection kit (Lonza, LT07-118) and MycoAlertTM Assay Control Set (Lonza, LT07-518). Cells were grown at 37°C with 5% CO2.
Commonly misidentified lines (See ICLAC register)	No commonly misidentified cell lines were used.

Human research participants

Policy information about studies involving human research participants

Population characteristics	As an opportunistic collection of data, the participants in this study were not selected based on age, gender, or genotypic information. Individuals in the gnomAD dataset have an average age of 53.6 years and 45.8% are female. Individuals were mainly recruited from complex disease studies. Any individuals with severe pediatric disease were removed. In the UK Biobank, individuals have an average age of 56.8 years and 54.4% are female. 23andMe individuals have an average age of 48.8 years and 54.5% are female.
Recruitment	Participants were either customers of the personal genetics company 23andMe, Inc., who consented to participate in research and answer survey questions online, were consented participants of the UK Biobank, or were aggregated as part of the genome aggregation database (gnomAD). Individuals from 23andMe were identified based on their genotyping results and as such were not specifically recruited. Therefore they share the same selection biases such as under representation of certain ethnic and socioeconomic groups relative to the general population. This limits our ability to make any firm conclusions in those groups. Similar biases exist within the gnomAD and UK Biobank datasets which represents secondary use of available sequencing.
Ethics oversight	23andMe - Participants provided informed consent and participated in the research online, under a protocol approved by the external AAHRPP-accredited IRB, Ethical & Independent Review Services (E&I Review).<br>gnomAD - We have complied with all relevant ethical regulations. This study was overseen by the Broad Institute's Office of Research Subject Protection and the Partners Human Research Committee. Informed consent was obtained from all participants.<br>UKBB - This work was done as part of approved UKBB project #42890

Note that full information on the approval of the study protocol must also be provided in the manuscript.
