# Supplementary figures and images for: The effect of LRRK2 loss-of-function variants in humans
Source: Nat Med. 2020 May 27;26(6):869–77. doi: 10.1038/s41591-020-0893-5 (PMC7303015; doi:10.1038/s41591-020-0893-5)

Unprocessed Immunoblots for Figure 2

(a) LRRK2

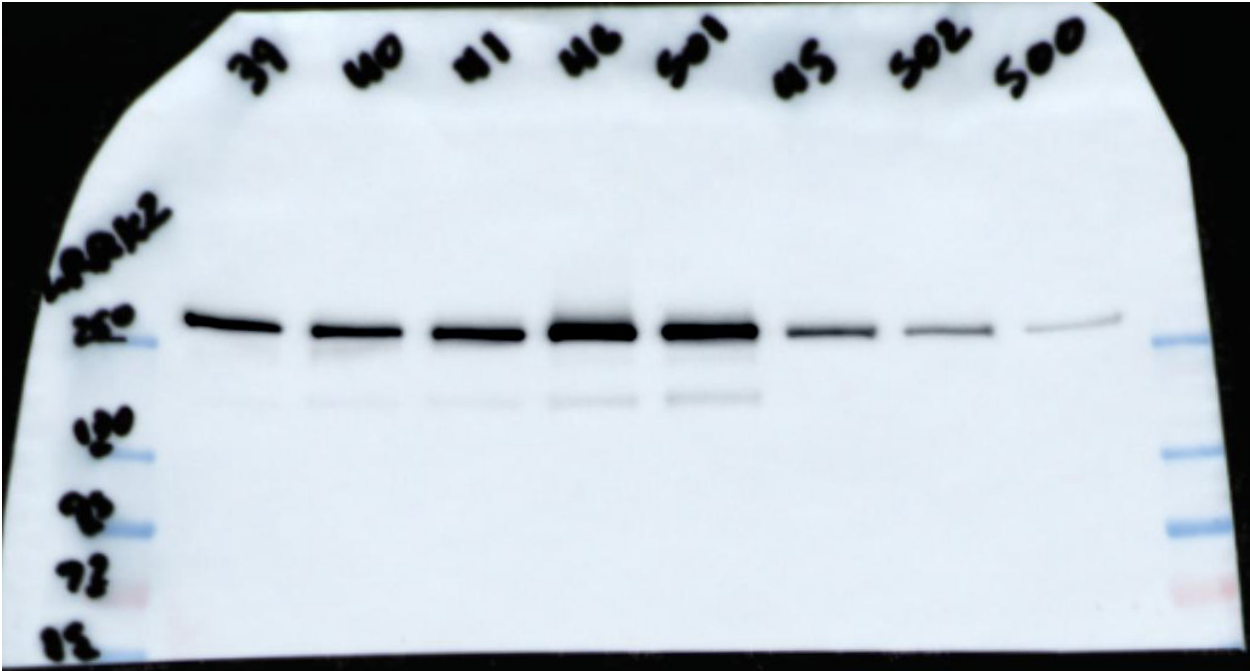

GAPDH

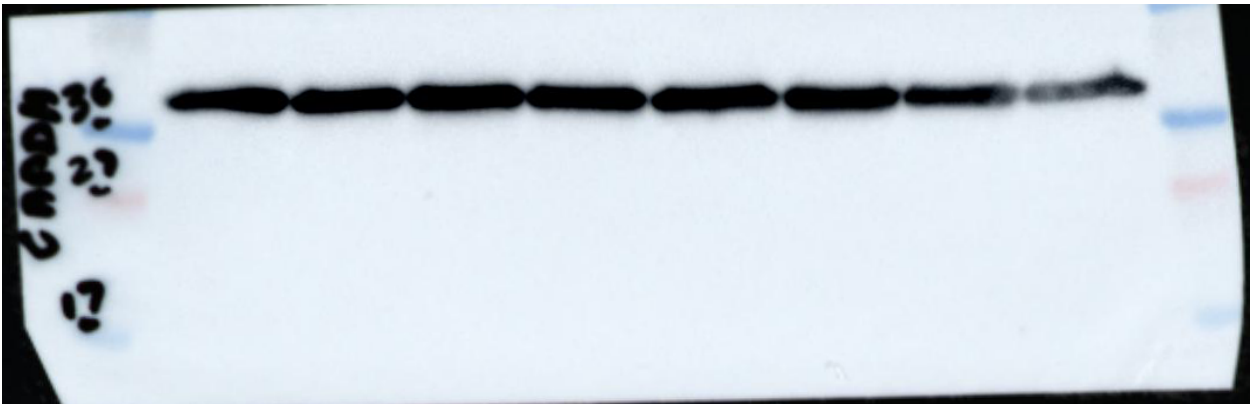

(b) LRRK2

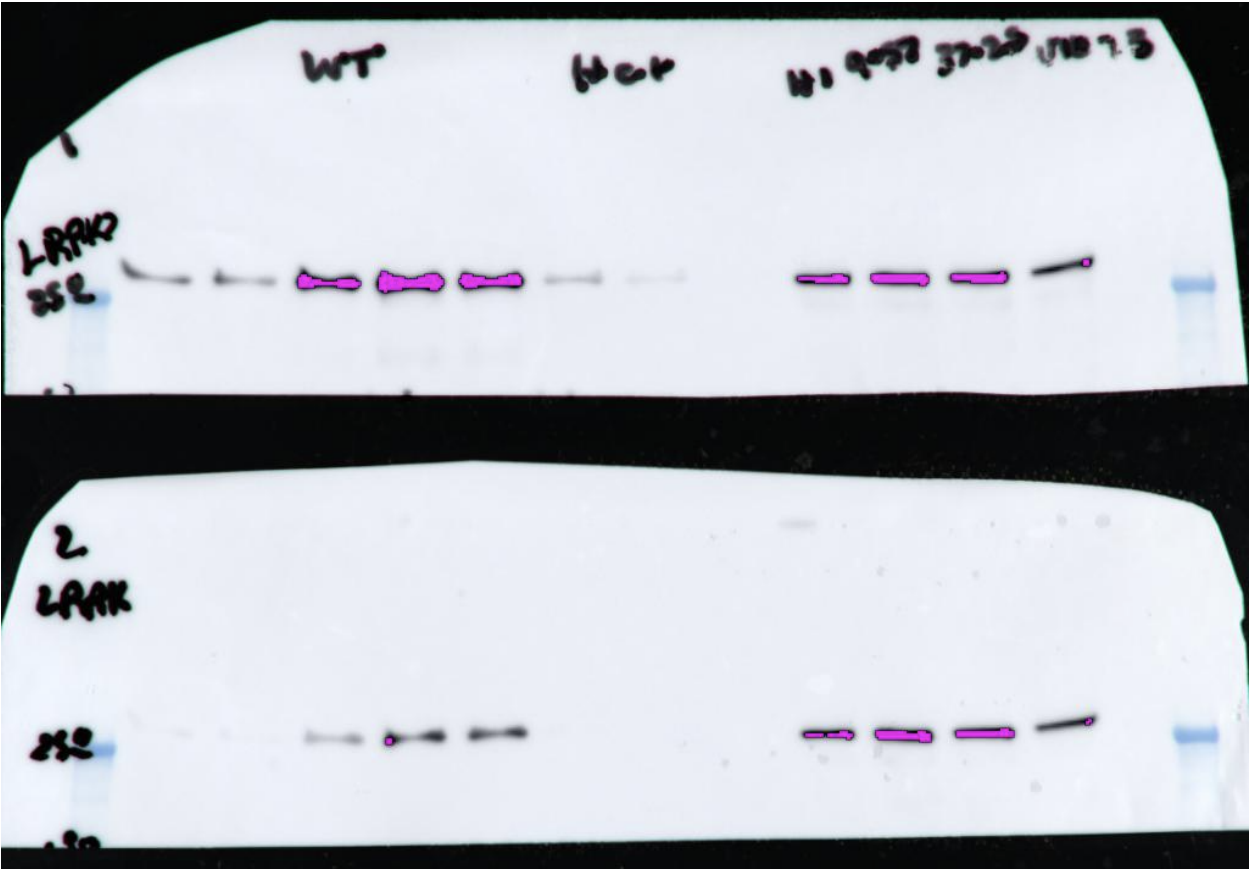

Alpha-actinin

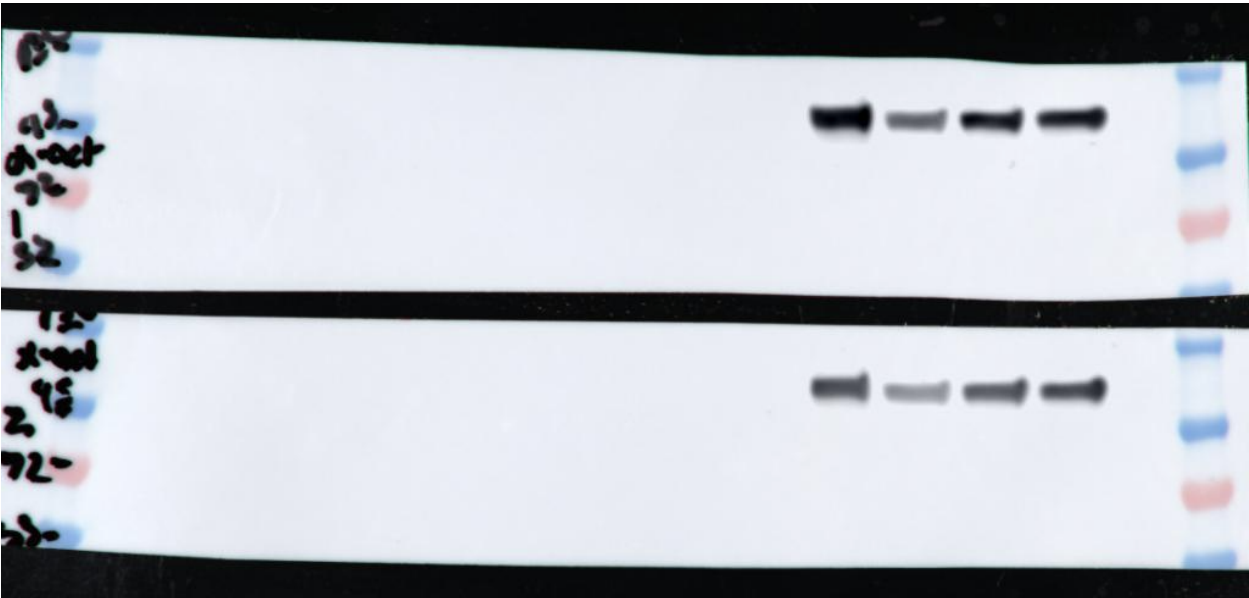

GAPDH

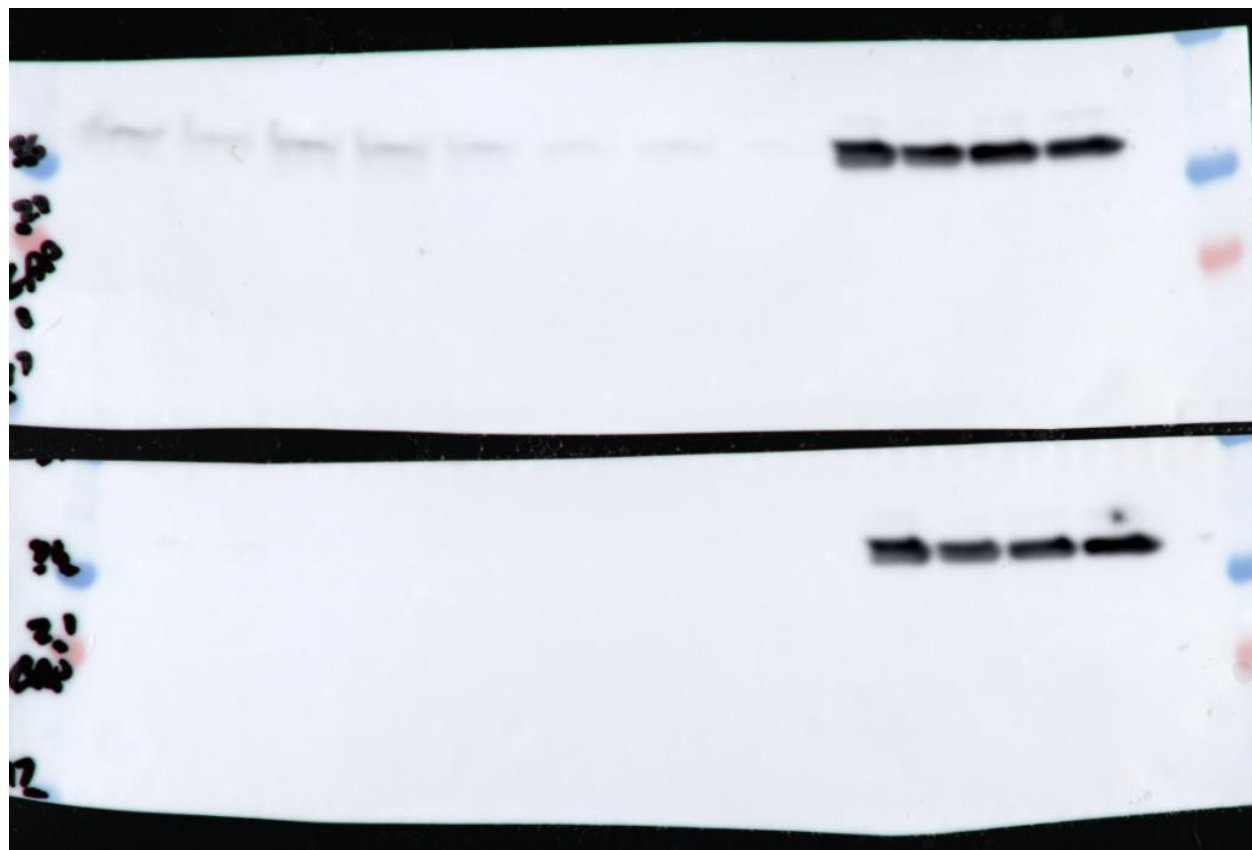

Supplement: Source Data Fig. 2 — Unprocessed immunoblots for Fig. 2. [file 41591_2020_893_MOESM4_ESM.pdf]
